# Transcriptional response to metal starvation in the emerging pathogen *Mycoplasma genitalium* is mediated by Fur-dependent and –independent regulatory pathways

**DOI:** 10.1080/22221751.2019.1700762

**Published:** 2019-12-20

**Authors:** Carlos Martínez-Torró, Sergi Torres-Puig, Marta Monge, Lucía Sánchez-Alba, Miguel González-Martín, Marina Marcos-Silva, Alex Perálvarez-Marín, Francesc Canals, Enrique Querol, Jaume Piñol, Oscar Q. Pich

**Affiliations:** a Institut de Biotecnologia i Biomedicina and Departament de Bioquímica i Biologia Molecular, Universitat Autònoma de Barcelona, Barcelona, Spain; b Vall d'Hebron Institute of Oncology (VHIO), Barcelona, Spain; c Biophysics Unit, Department of Biochemistry and Molecular Biology, School of Medicine, Universitat Autònoma de Barcelona, Barcelona, Spain

**Keywords:** *Mycoplasma genitalium*, emerging STI pathogen, novel therapeutic targets, metal acquisition systems, Ferric uptake regulator, Histidine-rich proteins, metallome

## Abstract

Transition metals participate in numerous enzymatic reactions and they are essential for survival in all living organisms. For this reason, bacterial pathogens have evolved dedicated machineries to effectively compete with their hosts and scavenge metals at the site of infection. In this study, we investigated the mechanisms controlling metal acquisition in the emerging human pathogen *Mycoplasma genitalium*. We observed a robust transcriptional response to metal starvation, and many genes coding for predicted lipoproteins and ABC-transporters were significantly up-regulated. Transcriptional analysis of a mutant strain lacking a metalloregulator of the Fur family revealed the activation of a full operon encoding a putative metal transporter system and a gene coding for a Histidine-rich lipoprotein (Hrl). We recognized a conserved sequence with dyad symmetry within the promoter region of the Fur-regulated genes. Mutagenesis of the predicted Fur operator within the *hrl* promoter abrogated Fur- and metal-dependent expression of a reporter gene. Metal starvation still impelled a strong transcriptional response in the *fur* mutant, demonstrating the existence of Fur-independent regulatory pathways controlling metal homeostasis. Finally, analysis of metal accumulation in the wild-type strain and the *fur* mutant by ICP-MS revealed an important role of Fur in nickel acquisition.

## Introduction

Urethritis or inflammation of the urethra is a medical condition intimately related to sexually transmitted infections (STI). Non-gonococcal urethritis is the most common treatable sexually transmitted syndrome in men [1,2] and it is frequently associated with infections by *Chlamydia trachomatis* and *Mycoplasma genitalium*. *M. genitalium* is an emergent human pathogen that has been also associated with several syndromes in women including cervicitis, endometritis and pelvic inflammatory disease [3,4]. Appreciation of the significance of *M. genitalium* in human disease has been hampered by *in vitro* culture limitations and lack of commercial molecular-based tests for rapid detection of this bacterium [5]. Prevalence of *M. genitalium* infections range from 0.7% to 3.3% in the general population, but this percentage increases dramatically in specific populations at high risk of STI [2,6]. In this sense, *M. genitalium* is more prevalent than any other bacterial STI in HIV-positive men who have sex with men [7].

Remarkably, numerous studies indicate that *M. genitalium* is rapidly developing resistance to all current standard antibiotic treatments, and is bound to become a major untreatable STI [5,8]. Supporting this idea, prevalence of mutations associated with azithromycin resistance, the first-line therapy to treat *M. genitalium* infections, rises up to 50% in particular contexts. Moreover, recent studies document a rapid emergence of mutations associated with resistance to the fourth-generation fluoroquinolone moxifloxacin, which is used as the second-line therapy to treat *M. genitalium* infections in Europe. Compounding this problem is the observation made years ago that 60% to 80% of *M. genitalium* infections fail to respond to tetracyclines, which are still used in many parts of the world as primary treatment for non-gonococcal urethritis. Overall, treatment of *M. genitalium* is increasingly challenging with resistant cases requiring costly drugs, which often have limited availability [9,10]. In consequence, there is an urgent need to identify novel therapeutic targets and to develop alternative antimicrobial strategies to combat *M. genitalium* infections.

Metal acquisition systems are fundamental components of bacterial pathogens as they are essential to compete with the host for available limited resources. Accordingly, bacteria have developed diverse strategies to acquire metals within the host. For example, many bacteria have the ability to scavenge iron from storage proteins found in the circulatory system, such as haemoglobin and transferrin, or on mucosal surfaces such as lactoferrin. To this end, they synthesize and secrete molecules with high affinity for metals, known as metallophores, to confiscate these otherwise inaccessible essential elements [11]. On the other hand, some bacteria produce potent cytotoxins meant to destroy target host cells and release the intracellular content to increase nutrient availability. Interestingly, the expression of many of these cytolysins, including the shiga toxin from *Shigella dysenteriae*, the diphtheria toxin from *Corynebacterium diphtheriae*, the exotoxin A from *Pseudomonas aeruginosa* or the *cagA* cytotoxin of *Helicobacter pylori*, responds to changes in iron availability [12,13]. Additionally, it has been shown that *H. pylori* exploits the *cagA* cytotoxin to perturb host trafficking systems and increase metal supply at the sites of infection [14,15]. Therefore, there is a tight connection between metal acquisition systems and bacterial virulence.

While essential for survival, freely available metals are toxic both for the host and the pathogen. Therefore, expression of metal acquisition systems is usually controlled by regulatory proteins that sense and coordinate the response to changes in metal availability. Transcriptional regulators of the Fur (Ferric Uptake Regulator) family are widespread in bacteria and they control the acquisition and storage of transition metals [16]. So far, Fur regulators have been shown to coordinate gene expression in response to changes in iron (Fur), zinc (Zur), nickel (Nur) and manganese (Mur) availability [17]. In the presence of metal, Fur proteins bind to specific DNA sequences, known as Fur boxes, located within the promoter region of target genes and block RNA polymerase binding. Conversely, when metals are scarce, Fur proteins are unable to bind their DNA targets and transcription of the regulated genes is de-repressed. Remarkably, inactivation of the *fur* locus often leads to important colonization defects and attenuated virulence in many pathogenic bacteria, including *H. pylori*, *Staphylococcus aureus*, *Campylobacter jejuni*, *Listeria monocytogenes*, *Actinobacillus pleuropneumoniae*, *Bacillus cereus* and *Vibrio cholerae* [18]. However, so far, only few studies have addressed the response to iron starvation in mycoplasmas [19,20] and the mechanisms controlling metal homeostasis in these bacteria remain essentially unknown.

In this study, we demonstrate that a metalloregulator of the Fur family is involved in the regulation of nickel acquisition in *M. genitalium*. A Histidine-rich lipoprotein and an energy coupling factor-type ABC transporter system arise as the main nickel acquisition systems of this bacterium. In addition, we reveal the existence of Fur-independent regulatory pathways controlling metal homeostasis in this pathogen. The results of this study may facilitate the development of novel therapeutic strategies to control *M. genitalium* infections based on molecules targeting the metal acquisition systems identified.

## Materials and methods

### Strains and culture conditions

*Mycoplasma genitalium* strains were grown in SP-4 medium in tissue culture flasks at 37°C in a 5% CO_2_ atmosphere. SP-4 plates were prepared supplementing the medium with 0.8% agar (BD). Puromycin (3 µg ml^−1^), tetracycline (3 µg ml^−1^) or chloramphenicol (17 µg ml^−1^) were used for mutant selection. *Escherichia coli* strain XL1-Blue was used for cloning and plasmid propagation. The strain was grown in LB broth or LB supplemented with 1.5% agar, 100 µg ml^−1^ ampicillin, 40 µg ml^−1^ X-Gal and 24 µg ml^−1^ Isopropyl β-D-1-thiogalactopyranoside (IPTG), when necessary. All *M. genitalium* strains used in this study are listed in Supplemental Table S1. Additionally, a full description of the procedure used to construct the different mutants is provided as Supplemental Information.

### Plasmids and primers

All plasmids and primers used in this work are listed in Supplemental Tables S2 and S3. In addition, a complete description of all plasmids used in this study is provided as Supplemental Information.

### DNA manipulation

Plasmid DNA was obtained using Fast Plasmid Mini kit (5Prime) or GeneJET Plasmid Miniprep Kit (Thermo Fisher Scientific). PCR products were purified from agarose gels using Nucleospin Gel and PCR Clean-up kit (Macherey-Nagel) and digested with the corresponding restriction enzymes (Thermo Fisher Scientific) when necessary. Plasmids for electroporation of *M. genitalium* were obtained using the GenElute HP Midiprep Kit (Sigma).

### Transformation and screening for mutants

Transformation of *M. genitalium* was achieved by electroporation as previously described [21] using either an ECM 630 (BTX) or a Gene Pulser Xcell (Bio-Rad) electroporator. We used 30 µg of plasmid DNA to generate mutants by allelic exchange and 10 µg for transposon mutants. Screening for mutants was performed using cell lysates as template for PCR and sequencing reactions, as previously described [22].

### Sequencing reactions

DNA sequencing reactions were performed with the BigDye® v3.1 Cycle Sequencing kit using 2.5 µl of *M. genitalium* lysate as a template, following manufacturer’s instructions. All reactions were analysed in an ABI PRISM 3130xl Genetic Analyzer at the Servei de Genòmica i Bioinformàtica (UAB).

### RNA-Seq analysis

Mid-log phase cultures of *M. genitalium* were scraped off in 1 mL of fresh SP-4 and reinoculated in two new 25 cm^2^ tissue culture flasks with fresh SP-4 medium for 6 h. Then, cells were lysed, and total RNA was extracted using the miRNeasy Mini Kit (Qiagen). When necessary, cultures were treated with the iron chelator 2,2-bypiridyl (1 mg ml^−1^) (Sigma-Aldrich) for 1 h prior to cell lysis to generate an iron limited environment.

RNA libraries were prepared with TruSeq Stranded Total RNA Library Prep Kit (Illumina) and analysed using a HiSeq 3000 System (Illumina) at the Genomics Unit from the Centre for Genomic Regulation (CRG), Barcelona. cDNA clusters were immobilized in sequencing lanes of 2 × 50 reads. Reverse and complementary was computed for sequences coming from Read1 primer. Data analysis and sequence alignment was performed using Bowtie2 [23] in the End-to-End mode and Forward-Forward paired-ends. Sequences were piled up using SAMtools [24] with no limited set to the number of sequences in the alignment. Counts in the different ORFs were performed with a standalone version of featureCounts programme [25] without counting the multi-mapping reads and disabling multi-overlapping reads.

Counted features were then submitted to the R/Bioconductor package DESeq2 [26] for statistical analysis. DESeq2 analysis used a parametric fitType and a zero-mean normal prior on the non-intercept coefficients. Data were sorted by log2 fold change and statistical significance was set at the common threshold of *P*-value < 0.05. Three independent biological repeats of each strain or condition were analysed.

### qRT-PCR analysis

RNA was extracted from mid-log phase cultures of *M. genitalium* using the RNAqueous Kit (Thermo Fisher Scientific) and then treated with Turbo DNase (Thermo Fisher Scientific) following the manufacturer’s instructions. When necessary, cultures were treated with the iron chelator 2,2-bypiridyl (1 mg ml^−1^) (Sigma-Aldrich) for 1 h before the cell lysis to create a transition metal limited environment. Reverse transcription was performed with iScript Reverse Transcriptase (Bio-Rad) and random primers as previously described [21]. Primers used for qPCR are listed in Supplemental Table S3 and they were designed using Primer3 software. qPCR was performed with iTaq polymerase (Bio-Rad) and SYBR green in CFX96 or CFX384 PCR instruments (Bio-Rad). Relative gene expression was calculated using the Pfaffl method [27]. Differential gene expression was judged based on the common arbitrary 2-fold cutoff. Data presented in the manuscript correspond to the analysis of RNAs isolated from three independent biological repeats.

### Primer extension

Primer extension analyses were performed with 20 µg of total RNA as previously described [21]. RNA was extracted using the RNAqueous kit (Thermo Fisher Scientific) and treated with Turbo DNase (Thermo Fisher Scientific). Primers used for this technique (Supplemental Table S3) were labelled with the 6-FAM fluorescent dye at the 5’ end. Fragments were separated using an ABI PRISM 3130xl Genetic Analyzer (Servei de Genòmica i Bioinformàtica, UAB) and then analysed using PeakScanner v1.0 software (Thermo Fisher Scientific).

### Differential proteomic analysis by 2D gel electrophoresis DIGE

Total protein for 2D-DIGE analysis was extracted as previously described [28]. Briefly, cells were washed three times in PBS and lysed in lysis solution (8 M urea, 2 M thiourea, 2.5% CHAPS, 2% ASB-14, 60 mM DTT, 40 mM Tris-HCl pH 8.8 and protease inhibitor cocktail) prior to sonication. Protein solutions were further purified by a modified TCA-acetone precipitation (2D-CleanUp Kit, GE Healthcare) and, finally, dissolved in the DIGE labelling buffer (8 M urea, 4% w/v CHAPS, 30 mM Tris pH 8.0). Protein concentration was determined using the Bio-Rad RCDC Protein Assay (Bio-Rad, UK), following manufacturer’s instructions. Triplicate protein samples of each of the strains were labelled using Cy3 or Cy5 cyanine dyes and separated in three two-dimensional electrophoresis gels as previously described [28]. Fluorescence images of the gels were obtained on a Typhoon 9400 scanner (GE Healthcare). Cy3 and Cy5 images were scanned at excitation/emission wavelengths of 532/580 nm and 633/670 nm, respectively. Image analysis and quantification of relative protein abundances were performed using SameSpots (Nonlinear Dynamics, UK).

### Protein identification by mass spectrometric analysis

Protein spots were excised from the gel using an automated Spot Picker (GE Healthcare). In-gel trypsin digestion was performed using autolysis stabilized trypsin (Promega, Madison, USA). Protein digests were analysed by liquid chromatography-mass spectrometry analysis on a Maxis Impact Q-TOF spectrometer (Bruker, Bremen), coupled to a nano-HPLC system (Proxeon, Denmark). Samples were evaporated and dissolved in 5% acetonitrile, 0.1% formic acid in water, and concentrated on a 100 μm ID, 2 cm Proxeon nanotrapping column and then loaded onto a 75 μm ID, 25 cm Acclaim PepMap nanoseparation column (Dionex). Chromatography was run using a 0.1% formic acid - acetonitrile gradient (5–35% in 30 min; flow rate 300 nL/min). The column was coupled to the mass spectrometer inlet through a Captive Spray (Bruker) ionization source. MS acquisition was set to cycles of MS (2 Hz), followed by Intensity Dependent MS/MS (2–20 Hz) of the 20 most intense precursor ions with an intensity threshold for fragmentation of 2500 counts, and using a dynamic exclusion time of 0.3 min. LC-MSMS data was analysed using the Data Analysis 4.0 software (Bruker). Proteins were identified using Mascot (Matrix Science, London UK) to search against the mycoplasma genitalium proteins in the UniProt/SwissProt database. MS/MS spectra were searched with a precursor mass tolerance of 10 ppm, fragment tolerance of 0.05 Da, trypsin specificity with a maximum of 2 missed cleavages, cysteine carbamidomethylation set as fixed modification and methionine oxidation as variable modification. Significance threshold for the identifications was set to *p* < 0.05, minimum ions score of 20.

### Phase contrast and fluorescence microscopy

*M. genitalium* cells were grown on 8-well IBIDI chamber slides for 16 h, washed three times with PBS and visualized on a Nikon Eclipse TE 2000-E microscope. Phase contrast and TRITC epifluorescence images were captured with a Digital Sight DS-SMC Nikon camera using the NIS-Elements BR Software. ImageJ was used to analyse fluorescence images.

### Inductively coupled plasma mass spectrometry (ICP-MS)

*M. genitalium* strains were grown in 175 cm^2^ tissue culture flasks with SP-4 medium until mid-log phase. Then, attached mycoplasmas were scraped off in 1 mL of fresh medium and used to start a 1L suspension culture. After 5 days of incubation at 37°C at 100 rpm, cells were harvested by centrifugation and washed with PBS. In some experiments, PBS washes were performed in the presence of 0.5 mM EDTA to remove extracellular metals as suggested by Williams and coworkers [29]. Next, pellets were dried overnight at 70°C on a Ultrawave microwave (Milestone) and digested with HNO_3_ (Merck). Finally, the sample amount was determined using a XPE205DR analytic balance (Metler Toledo) and analysed in a 7500ce ICP-MS instrument (Agilent).

## Results

### Global response of *M. genitalium* to iron starvation

**Figure 1.**
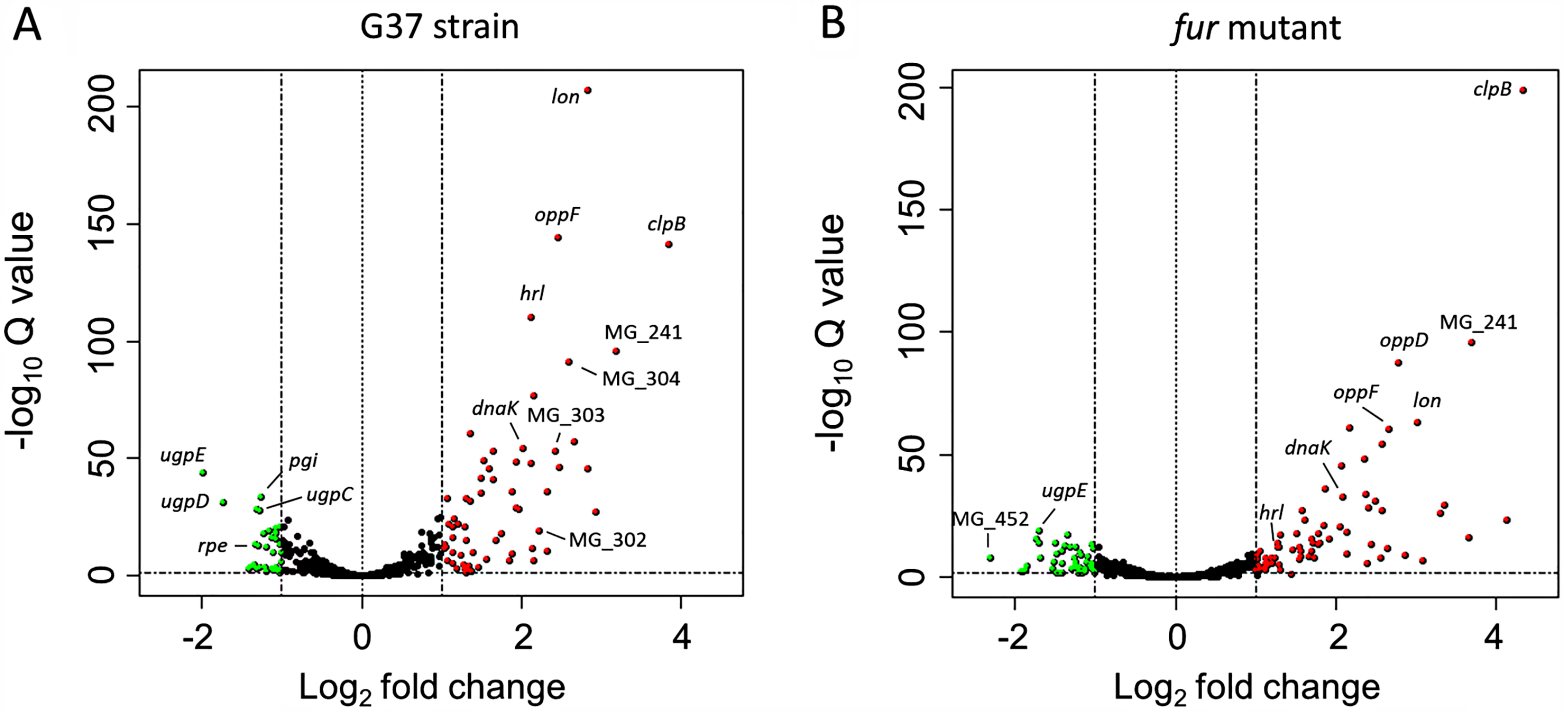
Volcano plot of transcriptional differences in *M. genitalium* upon shock with the iron chelator 2,2’-bipyridyl (DPP) identified by RNA-Seq analysis. Each spot represents a single gene. Red and green colours indicate activation and repression, respectively. Cut off for biological significance was set to log_2 _= 1.

We conducted a genome-wide RNA-Seq analysis to investigate transcriptional changes following metal depletion in *M. genitalium*. To this end, we shocked the cells with the metal chelator 2,2’-bipyridyl (DPP) and compared transcription to that of *bacteria* cells grown under routine culture conditions. DPP has been shown to bind iron with high affinity, although it can also coordinate other transition metals, especially when used at high concentrations. Our analysis revealed a robust response to metal starvation, with more than eighty differentially expressed genes belonging to diverse functional categories **(** Figure 1 and Supplemental Table S4). Remarkably, we observed a pronounced up-regulation of the molecular chaperone *dnaK* (4-fold) and the protease genes *clpB* (14-fold) and *lon* (7-fold), which are important to preserve protein integrity in bacteria. Likewise, transcription of several genes involved in DNA repair including the excinuclease *uvrC* (MG_206), the ATP-dependent helicase *uvrD* (MG_244) or the recombinase *recA* (MG_339), was induced under metal-depleted conditions. Transcript levels of the HPr kinase gene *hprK* (MG_085) and the glycerol uptake facilitator *glpF* (MG_033), involved in the regulation of carbon metabolism and sugar transport, also increased upon metal depletion. In addition, we observed a marked activation of three genes, MG_149, MG_321 and MG_439, coding for predicted lipoproteins.

Of note, we found that clusters of genes with related functions were consistently co-regulated. For instance, transcription of an operon coding for the different subunits of a putative ABC-type metal transporter system (MG_304, MG_303 and MG_302) increased by 5-fold upon iron starvation. Similarly, genes coding for a putative oligopeptide ABC-type transporter system (*oppBCDF*) were up-regulated by 4-fold. Furthermore, transcription of the MG_241 and MG_242 genes, which are co-transcribed with the *uvrD* locus, was also activated 9- and 6-fold, respectively. These genes code for two proteins with two transmembrane segments, indicating a membrane-associated function.

On the other hand, transcription of an operon coding for an ABC transporter system involved in glycerol-3-phosphate uptake (*ugpCAE* genes) was down-regulated by 3-fold upon the shock with 2,2’-bipyridyl. In addition, metal starvation inhibited transcription of several genes related to carbohydrate metabolism, including the glycolytic enzymes glucose-6-phosphate isomerase (*pgi*, MG_111) and fructose bisphosphate aldolase (*fba*, MG_023). Similarly, we detected decreased transcript levels of the ribulose-phosphate 3-epimerase (*rpe*, MG_112) and the UTP-glucose-1-phosphate uridylyltransferase (*galU*, MG_453) genes. Therefore, metal depletion prompts an important remodelling of the metabolic flux in *M. genitalium*. Furthermore, we found that transcription of the stringent response regulator gene *relA* was also inhibited, which is consistent with a coordinated response to nutrient deprivation. In summary, the response of *M. genitalium* to metal starvation is both robust and diverse, and it likely involves several regulatory proteins and pathways.

### Role of Fur in the regulation of metal homeostasis in *M. genitalium*

**Figure 2.**
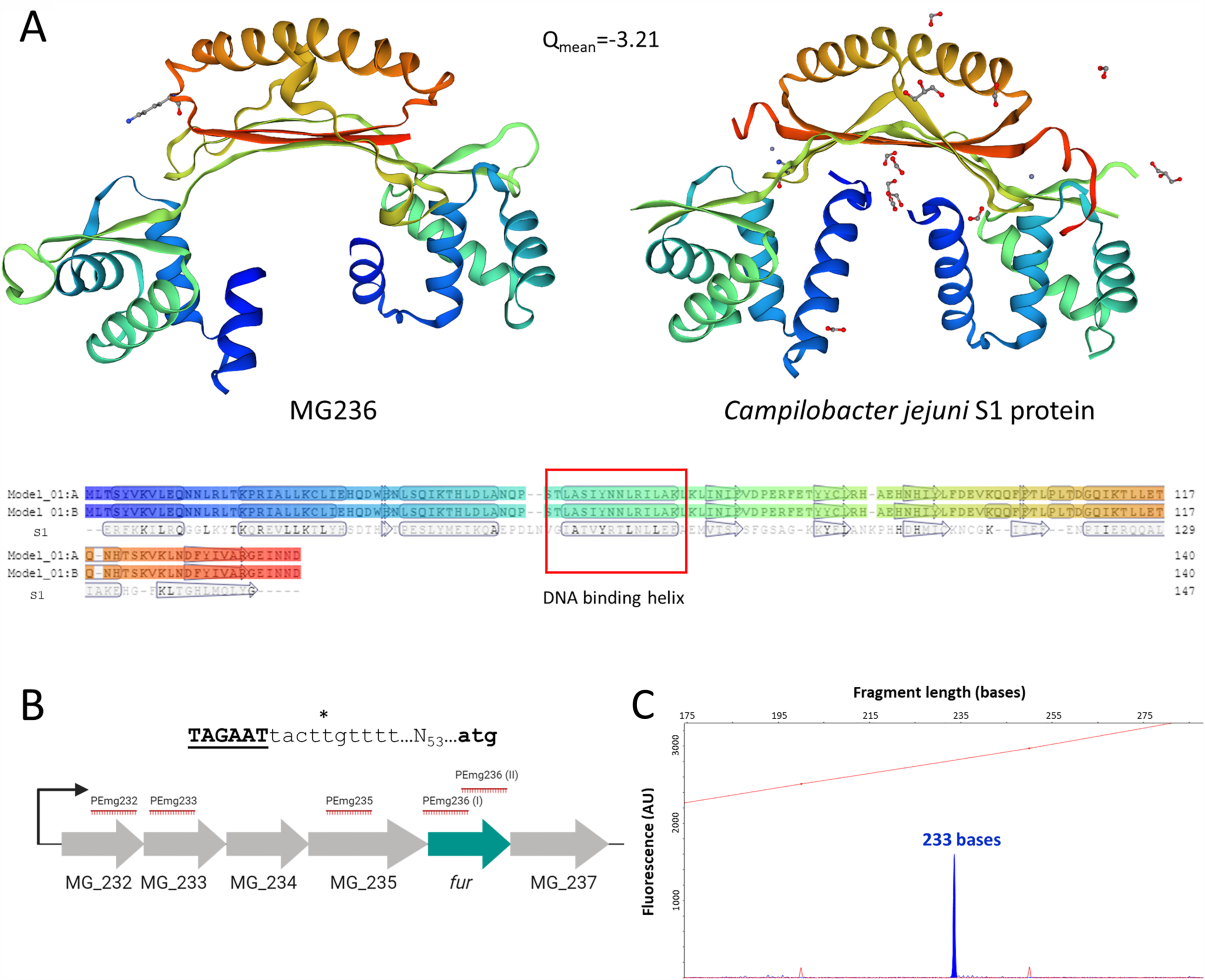
Homology of the *M. genitalium* MG236 protein to transcriptional repressors of the Fur family and determination of the MG_236 transcriptional start site (TSS). (A) SWISS-MODEL predicted 3D dimeric structure of MG236 (left) using *Campylobacter jejuni* fur homolog S1 dimer as a template (right) [66]. Below, model-template alignment is shown, highlighting residues with an exact match and predicted secondary structure using DSSP. (B) Scheme of the *M. genitalium* operon comprising the *fur* gene. The asterisk indicates the experimentally identified TSS. The predicted −10 box is shown in black and underlined. The translational start codon of the MG_232 gene is shown in black. The determination of the MG_236 TSS was achieved with primer PEmg232. Previous experiments with other primers (marked in red) did not reveal additional TSS. (C) Primer extension analyses to identify the MG_236 TSS. Electropherogram was generated with Peak Scanner v1.0 (Applied Biosystems) analysis software. Red peaks represent ROX size standards and primer extension product is indicated in blue (233 bases).

**Figure 3.**
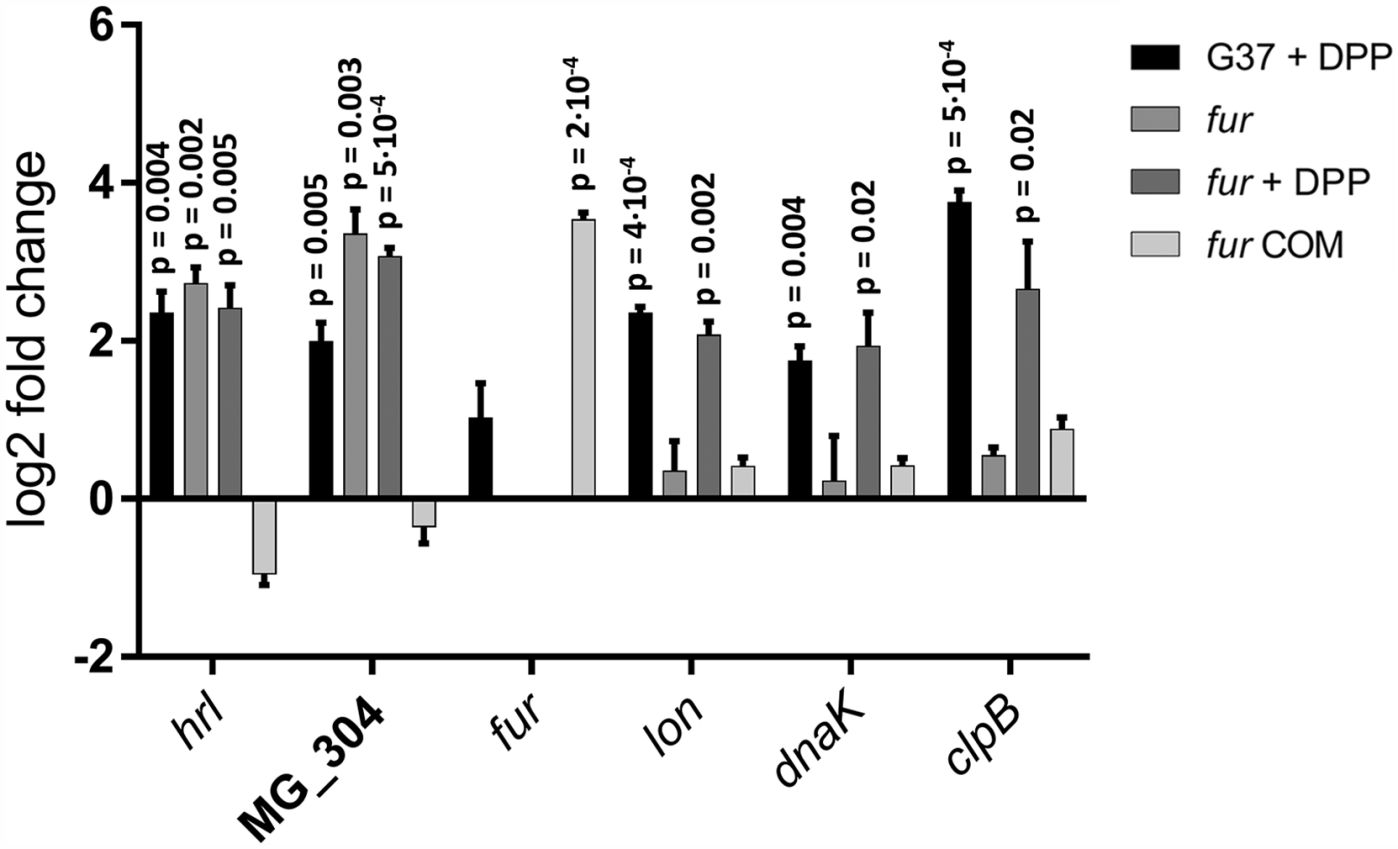
qRT-PCR analysis of *M. genitalium* G37, a *fur* mutant and its complemented strain derivative (*fur* COM) under standard growth conditions and upon the addition of 2,2’-bipyridyl. Bars represent the mean log2 fold-changes of three independent biological repeats. Statistical significance of mean fold-changes above the arbitrary cut off >1 for biological significance was assessed using a paired *t* test. *P*-values are located above each column when differences to the G37 strain are statistically significant (*p* < 0.05). The ectopic location of the *fur* gene in the complemented strain leads to a marked *fur* overexpression.

The MG236 protein of *M. genitalium* shows sequence homology to metalloregulators of the Fur family, which control metal homeostasis in many bacteria. Moreover, the predicted tridimensional structure of the MG236 protein using the SWISS-MODEL protein structure homology-modelling server [30] reveals a striking similarity to that of a Fur regulator from *C. jejuni* (Figure 2(A)). Our analyses indicate that *fur* in *M. genitalium* is co-transcribed with several genes coding for two ribosomal proteins (MG_232 and MG_233), the ribosomal-processing cysteine protease Prp (MG_234), the apurinic endonuclease IV Nfo (MG_235) and the unknown protein MG_237 (Figure 2(B)). To assess the possible implication of the *M. genitalium fur* homologue in the regulation of metal acquisition, we obtained a null mutant by allelic exchange. In the resulting *fur* mutant strain, the MG_236 gene is replaced by the tetracycline resistance marker (*tetM*). The terminator sequence of the *dnaK* gene, identified by the TranstermHP software [31], was placed after the *tetM* marker to prevent overexpression of the MG_237 gene. For control purposes, we also generated a complemented strain carrying an ectopic copy of the *fur* gene. Primer extension analyses revealed that transcription of *fur* in the wild-type strain was driven by a promoter located in the upstream region of the MG_232 gene (Figure 2(C)). Therefore, in the complemented strain, we imposed transcription of the transposon-encoded *fur* copy by the MG_232 promoter. However, all of the analysed clones overexpressed *fur* as compared to the wild-type strain (Figure 3). This is likely due to the presence of other promoters within the insertion site that increase transcription driven by the MG_232 promoter.

Transcriptional changes in the *fur* mutant under standard culture conditions were also assessed by RNA-Seq. We observed the activation (∼4-fold) of the histidine-rich lipoprotein gene (*hrl*, MG_149) and three genes (MG_302, MG_303 and MG_304) comprising a putative metal uptake system with homology to CbiMNQO transporter systems (Supplemental Table S5). As mentioned above, these genes were also up-regulated in the wild-type strain upon metal starvation. Reintroduction of an ectopic MG_236 copy to the *fur* mutant restored wild-type levels of transcription of the Fur-regulated genes (Supplemental Table S5). Transcript levels of the MG_237 gene were slightly lower in both the *fur* mutant and the complemented strain (∼2-fold), indicating some polar effects derived from the replacement of the *fur* gene by the *tetM* marker. qRT-PCR analysis confirmed the Fur-regulated expression of the *hrl* gene and the putative ABC-type metal transporter (Figure 3).

### Control of metal homeostasis by Fur-independent pathways

To get further knowledge on the regulatory mechanisms controlling metal homeostasis in *M. genitalium*, we investigated the existence of transcriptional changes in the *fur* mutant upon shock with the metal-chelator DPP. Despite the absence of the transcriptional regulator, a strong transcriptional response was still observed, and we identified up to one hundred differentially expressed genes upon metal depletion. **(** Figure 1 and Supplemental Table S6). We observed a good overlap between the response to metal starvation in the *fur* mutant and that in the wild-type strain. However, transcription of *hrl* and the *cbio* transporter operon (MG_302-MG_304) remained unchanged, indicating that activation of these genes in response to metal deprivation is entirely dependent on Fur. The existence of genes that respond to Fur and metal starvation, and many others that only respond to variations in metal availability, was confirmed by qRT-PCR analyses (Figure 3). Overall, our results demonstrate the existence of a global Fur-independent response to metal starvation in *M. genitalium*.

### Proteomic analysis of the *fur* mutant

**Figure 4.**
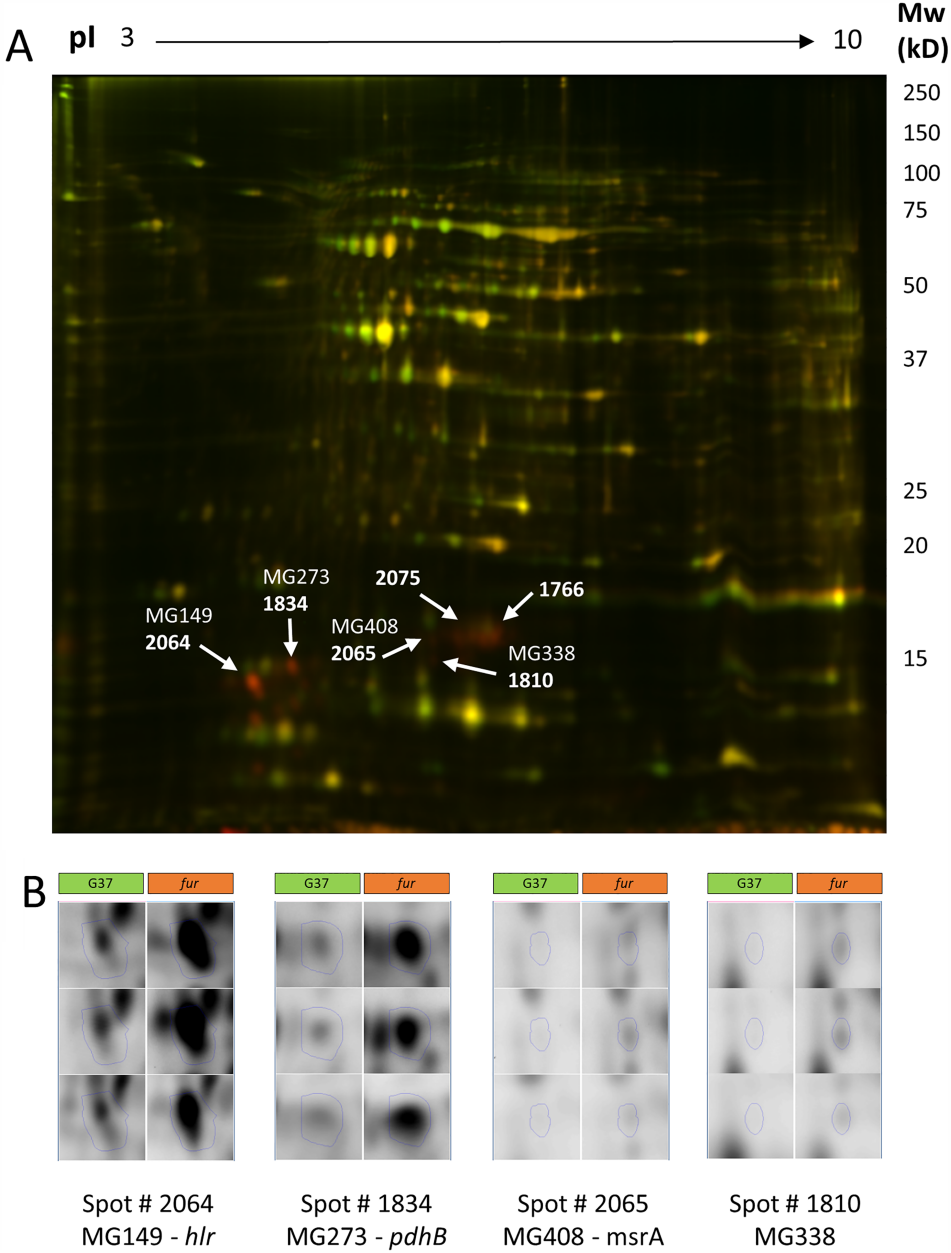
2D-DIGE analysis of the *M. genitalium* wild-type strain and a *fur* mutant. (A) Superimposed images in pseudocolour from Cy3 (green, WT proteins) and Cy5 (red, *fur* proteins) labelled samples run on a 2D-DIGE gel. The positions of some of the differential proteins are marked and labelled. (B) Fluorescence images corresponding to each of the 3 replicate samples of the WT and *fur* samples for some of the differential proteins identified.

Then, we wondered whether the transcriptional changes observed in the *fur* mutant were conserved at the protein level. To this end, we used 2D-DIGE and LC-MS to examine the differences in protein abundance between the wild-type strain and the *fur* mutant under standard culture conditions. (Figure 4). We identified sixteen protein spots with significant changes in expression and above the 2-fold arbitrary cutoff in the *fur* mutant compared to the wild type strain (Supplemental Table S7). In all cases, changes were consistent with higher expression levels in the *fur* mutant. Mass spectrometric analyses determined the presence of the Hrl (MG149) polypeptide in two spots (log2 fold change 2.22 and 2.60, respectively). Remarkably, the apparent molecular mass of this lipoprotein (16 kDa or below) was lower than the expected (32 kDa); this suggests a proteolytic cleavage at the centre of the protein. A similar observation was previously documented by Shimizu and coworkers [32].

The different subunits of the metal transporter system (MG304-MG303-MG302) were not identified in any of the analysed spots. However, in other spots, we also identified the presence of the MG338 lipoprotein (log2 fold change 2.45 and 2.60, respectively) and the Beta subunit of the Pyruvate dehydrogenase component E1 (MG273, PdhB). In addition, we identified the Methionine sulfoxide reductase A in three different spots (log2 fold change from 2.23–2.49) and the 30S ribosomal proteins S7 (log2 fold change 2.54) and S9 (log2 fold change 2.19 and 2.08, respectively), in one and two spots, respectively. Analysis of five protein spots did not produce any significant identification results.

### Identification of a regulatory element in Fur-regulated promoters

**Figure 5.**
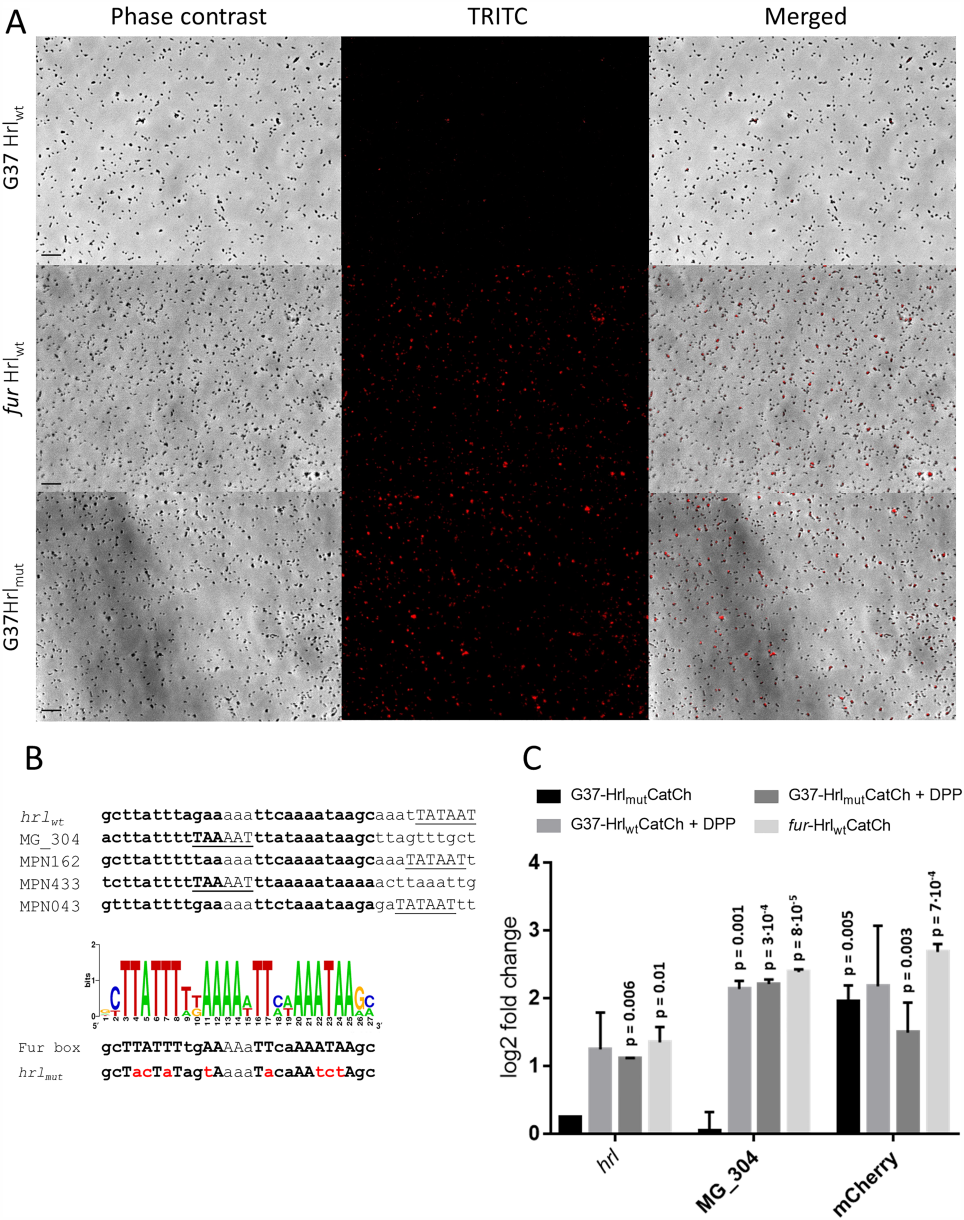
Expression of a gene reporter (*catcherry*) under the control of the *hrl* promoter of *M. genitalium*. (A) Fluorescence microscopy analysis. Each row contains a series of three images corresponding to the phase contrast, the Texas Red channel and the resulting overlay, respectively. (B) Identification of a putative Fur box sequence in *M. genitalium* and *M. pneumoniae*. MPN162, MPN433 and MPN043 are the respective homologs of MG_149 (*hrl*), MG_304 and MG_033 (*glpF*) in *M. pneumoniae.* Sequences in bold correspond to the conserved palindromic regions identified within the promoters of Fur-regulated genes of *M. genitalium* and *M. pneumoniae*. The graphic is a sequence logo generated with the conserved sequences. The putative −10 promoter elements are underlined. The overall height of each stack indicates the sequence conservation at that position. The scrambled promoter sequence used to generate the mutant strain characterized in panel 5A is located below the consensus Fur box sequence and the mutated residues are marked in red. (C) Transcriptional analysis by qRT-PCR. Bars represent the mean log2 fold-changes of three independent biological repeats. Statistical significance of mean log2 fold-changes above the arbitrary cut off >1 for biological significance was assessed with Student’s T test. Statistically significant values (*p* < 0.05) are indicated above the columns. Transcription of *hrl* and MG_304 was also analysed for control purposes.

*M. genitalium* Fur-regulated genes seem to be controlled by σ^70^-dependent promoters. Of note, we recognized a conserved sequence with dyad symmetry near the putative Pribnow boxes of these promoters (Figure 5(B)). To assess the possible contribution of this conserved sequence to Fur-regulation, we created a transcriptional fusion of the *hrl* promoter to the *mcherry* fluorescent marker. The resulting Hrl_wt_:CatCh cassette was introduced to *M. genitalium* by transposon delivery. All transformants analysed, designated G37-Hrl_wt_CatCh, exhibited marginal mCherry fluorescence (Figure 5(A)). This is consistent with low levels of expression of the mCherry marker in a wild-type background driven by the *hrl* promoter.

Then, we tested the expression of the Hrl_wt_:CatCh cassette in a *fur* mutant background. To this end, we first determined the insertion site of the minitransposon carrying the *mcherry* reporter in the different G37-Hrl_wt_CatCh mutants described above (Supplemental Table S8). Clone 1, with the minitransposon inserted in the intergenic region within the MG_339 and MG_340 genes, was selected for further analysis. In order to allow direct comparison of mCherry expression in different strain backgrounds, the Hrl_wt_:CatCh cassette was introduced into the *fur* mutant at the same chromosomal location as in clone 1 by homologous recombination. In the resulting *fur*-Hrl_wt_CatCh transformants, we observed mCherry fluorescence in all the clones analysed **(** Figure 5(A**))**. Using the same procedure, an *hrl* promoter with a scrambled Fur box was also introduced to the wild-type strain in the intergenic region within the MG_339 and MG_340 genes by homologous recombination. mCherry fluorescence was observed in both mutant backgrounds (Figure 5(A)). Overall, these data demonstrate the participation of the identified conserved sequence in Fur-regulation.

To obtain quantitative data, we assessed *mcherry* expression in these mutants by qRT-PCR (Figure 5(C)). As expected, transcript levels of the *mcherry* reporter were higher in the *fur* mutant than in the wild-type background (∼6-fold). Similarly, the presence of a mutated *hrl* promoter increased transcription of the *mcherry* reporter in a wild-type background (∼4-fold). In addition, we also tested the effect of the chelator 2,2’-bipyridyl on *mcherry* expression. We found that *mcherry* transcription was metal dependent in the wild-type strain (Figure 5(C)). Transcriptional analysis confirms the fluorescence data described earlier.

### Determination of the metallome of *M. genitalium*

**Figure 6.**
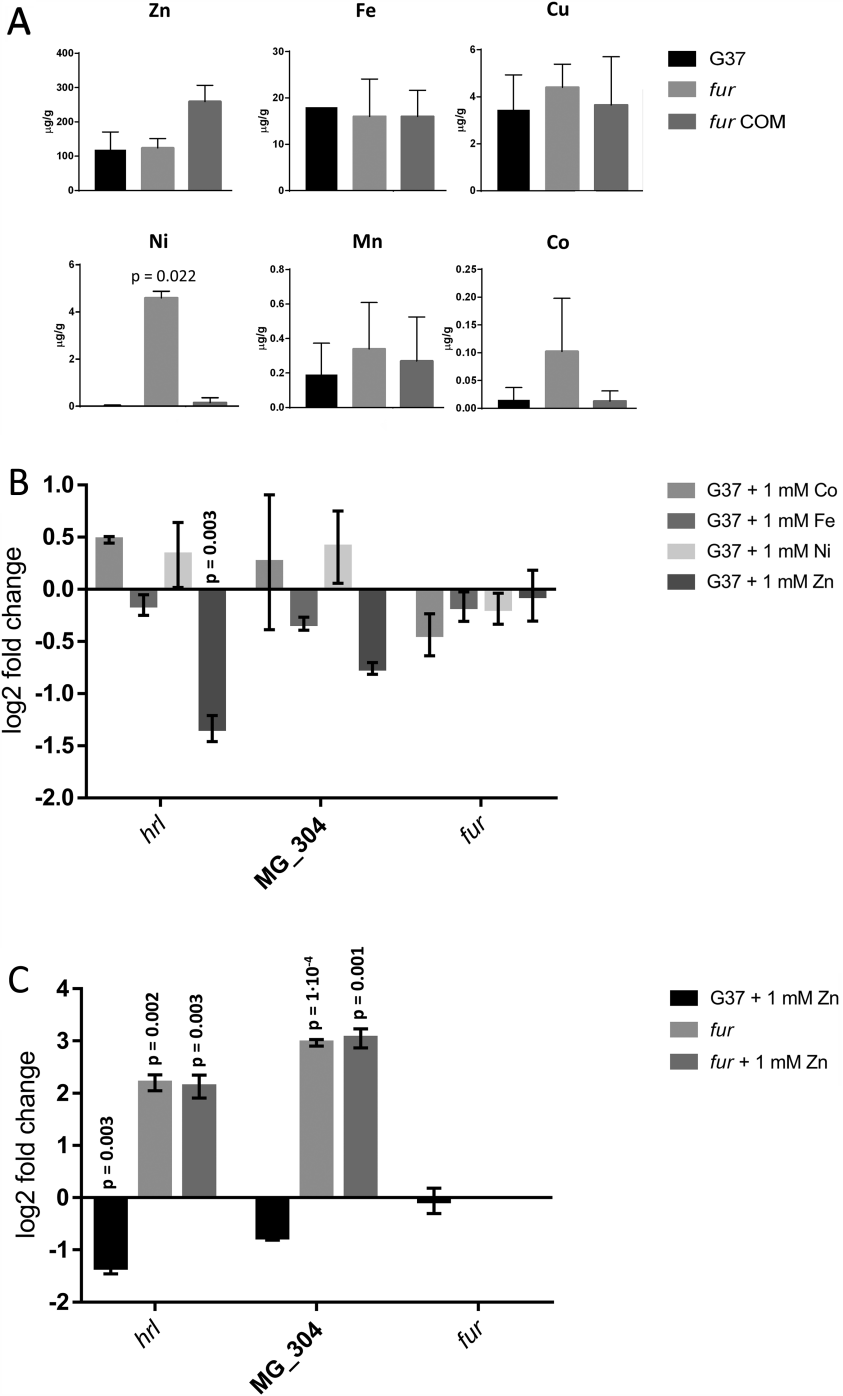
Acquisition and response to transition metals. (A) Analysis of metal accumulation in *M. genitalium* by inductively coupled plasma mass spectrometry (ICP-MS). Statistical significance was assessed with Student’s T test. Statistically significant values (*p* < 0.05) are indicated with their corresponding *p*-values. (B) qRT-PCR analysis of key Fur-regulated genes in the wild-type strain upon addition of 1 mM FeCl_2_, NiCl_2_, ZnCl_2_ or CoCl_2_. (C) qRT-PCR analysis of key Fur-regulated genes in the wild-type strain upon addition of 1 mM ZnCl_2._ Bars indicate the mean log2 fold-changes of three independent biological repeats. Statistical significance of mean log2 fold-changes above the arbitrary cut off >1 was assessed with Student’s T test. Statistically significant values (*p* < 0.05) are indicated above the corresponding columns.

The study of metal acquisition systems of *M. genitalium* prompted us to determine the metal content (metallome) of this pathogen under routine culture conditions. Culture medium of this bacterium is extraordinarily rich and it contains a significant amount of transition metals as supported by ICP-MS analysis (Supplemental Table S9). In pellets of the wild-type strain, zinc was the most abundant transition metal identified (∼100 µg g^−1^), followed by iron (∼20 µg g^−1^) and copper (∼3 µg g^−1^) (Figure 6(A)). Other metals such as nickel, manganese or cobalt were scarce or undetectable in our analysis. Treatment of the pellets with EDTA did not reveal any difference in metal accumulation. In contrast, cells from the *fur* mutant contained about 10-fold more nickel than the wild-type strain. In contrast, the intracellular metal content was effectively restored in the complemented *fur* mutant. Cobalt was also detected at higher levels in the *fur* mutant (∼5-fold), but this result varied largely among the different repeats. Therefore, our data indicate that Fur may be implicated in the regulation of nickel acquisition in *M. genitalium*.

### Effect of transition metals on Fur regulation

Finally, we evaluated the impact on Fur-regulation of an excess of some transition metals. We did not observe any transcriptional differences upon supplementation of the SP-4 medium with 1 mM CoCl_2_, FeCl_2_ or NiCl_2_. However, the addition of 1 mM ZnCl_2_ triggered a significant down-regulation of *hrl* and a slight decrease on MG_304 transcription (Figure 6(B)). Remarkably, these changes were Fur-dependent as an excess of zinc did no modify transcription of *hrl* or MG_304 in a *fur* background. Altogether, our data indicate that Fur could use zinc as a cofactor to regulate gene expression in *M. genitalium*.

## Discussion

Sequestration of micronutrients represents one of the first lines of defense of the host against bacterial infection. To counteract this defensive strategy, termed nutritional immunity [33], bacteria display a large repertoire of dedicated transporters to guarantee a sufficient supply of essential elements. Several years ago, Tryon and Baseman reported the ability of the respiratory pathogen *M. pneumoniae* to acquire human lactoferrin by a saturable and specific manner [34]. Lactoferrin is an iron-binding protein found at high concentrations in mucosal secretions that shows antibacterial activity both *in vitro* and *in vivo* [35]. However, the same study concluded that *M. genitalium* did no bind either lactoferrin or transferrin. Therefore, despite the clinical relevance of this emerging mucosal-surface pathogen, studies addressed to identify the metal acquisition strategies of *M. genitalium* are essentially non-existent.

Herein, we show that metal starvation elicits a broad transcriptional response in *M. genitalium*, suggesting that regulation of metal homeostasis in this bacterium involves different regulatory pathways. In this sense, we found that iron regulates transcription of several chaperone and protease genes under the control of the HrcA repressor, which coordinates the heat stress response in *M. genitalium* [36]. Similarly, transcript levels of *relA*, a major regulator of the nutrient-starvation response in bacteria [37], decrease upon metal depletion. On the other hand, we found a significant activation of an operon coding for an oligopeptide permease (Opp) transporter system (MG_077-MG_078-MG_079 and MG_080). Oligopeptide and dipeptide permeases have been found to be significantly up-regulated during iron starvation in other pathogenic bacteria [12,38] and they have been implicated in metal acquisition in *S. aureus* [39] and *E. coli* [40]. Moreover, some oligopeptide-binding proteins can accommodate haeme in its binding pocket [41], hence providing a new strategy to acquire iron from the host. In addition, the oligopeptide transport ATP-binding protein OppD has been shown to be critical for survival of the avian pathogen *Mycoplasma gallisepticum* within the respiratory tract [42], supporting the relevance of oligopeptide permeases in mycoplasma infection.

Additionally, our results also show that metal depletion activates transcription of the glycerol uptake facilitator gene (*glpF*). This finding is notable because glycerol metabolism is involved in hydrogen peroxide production in mycoplasmas and it represents a widespread virulence factor of these unique pathogens [43–45]. Therefore, an increased glycerol uptake capacity could enhance the cytotoxic potential of *M. genitalium* under metal limited conditions. On the other hand, we found that metal starvation inhibits transcription of several genes involved in carbohydrate metabolism. Moreover, we also observed decreased transcript levels of the gene coding for the Ribulose-phosphate 3-epimerase, an enzyme that has been shown to use zinc as a cofactor for catalysis along with cobalt and manganese [46]. Thus, our results indicate that metal deprivation prompts an important metabolic reprogramming in *M. genitalium*, with altered expression of several genes encoding for putative transporters and metabolic enzymes.

The MG_236 gene of *M. genitalium* codes for a Fur homologue, which are widespread metalloregulators controlling metal homeostasis in bacteria. Other human mycoplasmas including the respiratory pathogen *M. pneumoniae* (MPN329) and the STI pathogen *Mycoplasma penetrans* (MYPE1200), also contain putative Fur proteins. In a previous report, the MG_236 gene was classified as essential for *M. genitalium* growth under laboratory culture conditions [47]. However, we successfully deleted the MG_236 gene by allelic exchange using standard procedures, indicating an incomplete saturation of the transposon library characterized to assess gene essentiality. Transcriptional analysis of the *fur* mutant revealed the activation of a gene coding for a Histidine-rich lipoprotein (MG149) and a putative metal transporter system with homology to CbiMNQO uptake systems (MG302-MG303-MG304), which are putatively implicated in cobalt import in association with the cobalamin (vitamin B12) biosynthetic pathways. The *hrl* gene was previously shown to be markedly induced upon hyperosmotic shock in *M. genitalium* [48]. The overlap between osmotic stress and metal starvation in bacteria has been previously documented [49,50]. In the upstream region of the Fur-regulated genes, we recognized a conserved sequence with dyad symmetry with the consensus gcTTATTTtgAA-N3-TTcaAAATAAgc located near the predicted −10 promoter elements (Figure 5(B)). Proximity of the predicted Fur binding sites to key promoter elements is in agreement with the classic repressor role of Fur regulators, which bind to the DNA and prevent RNA polymerase binding. Of note, the putative Fur binding sites identified in *M. genitalium* differ considerably from the conventional Fur boxes originally described in *E. coli* [51,52]. However, divergent Fur operators have been identified in mycobacteria and streptomycetes [53]. Despite the presence of a Fur metalloregulator in *M. genitalium*, our analyses demonstrate that the transcriptional response to metal deprivation in this emerging pathogen is mainly Fur-independent.

In the respiratory pathogen *M. pneumoniae*, the presence in the genome of a pseudopalindromic sequence resembling the putative Fur boxes identified in this work was already pinpointed in two comprehensive transcriptional studies [20,54]. Candidate Fur binding sites in *M. pneumoniae* are located in the upstream region of the MPN162 (*hrl* homologue), MPN433 (*cbiO*) and MPN043 genes (*glpF*) (Figure 5(B)). Fur overexpression in *M. pneumoniae* induces transcriptional changes in genes containing the identified Fur boxes [54]. Unlike in *M. pneumoniae*, our results indicate that *glpF* expression is regulated by metal deprivation but not Fur in *M. genitalium*, which likely reflects the exquisite adaptation of these human pathogens to their respective infection niches.

On the other hand, we demonstrate that *M. genitalium* cells grown in a nutrient rich environment preferentially accumulate zinc, iron and copper, likely through constitutive metal transporters. This metal accumulation profile is similar to that described in *E. coli* by Zhao and co-workers [55]. According to this report, *E. coli* and *Enterococcus faecium* acquire preferentially iron, manganese and zinc, while nickel and cobalt are predominant in *S. aureus* and *Klebsiella pneumoniae*. Remarkably, ICP-MS analysis of a *M. genitalium fur* mutant revealed increased concentrations of nickel compared to the wild-type strain, establishing an important role for Fur in the acquisition of nickel in this urogenital pathogen. In addition, we found that high levels of zinc inhibit the expression of genes under the control of Fur, suggesting that this transition metal might regulate Fur function in *M. genitalium*. Indeed, crystal structures of Fur proteins from other bacteria have revealed coordination of zinc ions functioning as co-regulators to metal binding sites [56–58]. Furthermore, zinc coordination in the Fur homologue of *E. coli* has been proposed to be essential for dimerization, highlighting the importance of metals for structural maintenance and activity of Fur regulators [59]. This is also in agreement with a recent report, showing that thiolutin, a sulfur-containing antibiotic that has been used to chelate zinc[60], modifies transcription of Fur-regulated genes in *M. pneumoniae* [20]. Therefore, it is tempting to speculate that DPP treatment chelates zinc ions that are essential for Fur activity in *M. genitalium*, which leads to de-repression of the Fur regulon.

Nickel participates in different biological processes and they are considered essential micronutrients. Nickel is a cofactor of urease, Ni-Fe hydrogenases some superoxide dismutases, enzymes that do not seem to be present in *M. genitalium*. However, *M. genitalium* is a facultative anaerobic organism, which is relevant because many Ni^2+^-containing enzymes are active under low-oxygen conditions. Thus, we anticipate that *M. genitalium* encounters oxygen depletion during infection, which opens the possibility of the existence of unknown Ni^2+^-containing enzymes in this bacterium.

A search for conserved domains within Hrl using the MOTIF tool (www.genome.jp) reveals up to 13 hits, most of them belonging to known proteins involved in nickel, cobalt and zinc transport such as RcnA, ZnuA, CbtA, ZntC, CzcD or Zip (Supplemental Figure S1). For example, RcnA belongs to the Nickel/Cobalt Transporter (NicO) Family and it is believed to catalyze Co^2+^ and Ni^2+^ efflux in *E. coli* [61]. As mentioned earlier in this text, the putative metal transporter regulated by Fur in *M. genitalium* shows homology to CbiMNQO uptake systems [62]. The CbiMNQO protein complex belongs to the ECF (Energy Coupling Factor) subtype of ABC transporters, which are composed of two ATP-binding cassette ATPases (EcfA-MG304 and EcfA′-MG303) and a transmembrane coupling subunit (EcfT-MG302). These three core ECF proteins should interact with cognate substrate-binding subunits (EcfS), which confer ligand specificity. In *M. genitalium*, no *ecfS* gene has been identified in the vicinity of the metal responsive ECF-type operon identified in this study. Therefore, it is tempting to speculate that Hrl may function as a substrate-binding subunit of the CbiMNQO-like uptake system. Alternatively, although metallophores have not been described in mycoplasmas, Hrl may exhibit metallophore-like activity and cooperate with a yet to be identified EcfS protein to facilitate nickel uptake. Histidine-rich proteins are present in other mycoplasma species, where they are usually annotated as ZIP zinc transporter proteins. Experiments are under way to establish the exact role of Hrl and the CbiMNQO-like transporter in metal uptake.

Given the tight link between metal acquisition systems and virulence, Fur-regulated proteins constitute good therapeutic candidates for drug development. Transporters from the ECF family, which are not present in humans, have been proposed as potential antimicrobial targets. Assessment of the likelihood to find a selective, low-molecular weight molecule that binds with high affinity to the target, which is usually referred to as druggability assessment, identified up to twelve druggable pockets within a reference ECF transporter system [63]. An alternative approach to prevent metal acquisition could be either the inhibition of binding of the S-components to the ECF module or the inhibition of their ability to bind metals. In this sense, compounds inhibiting Hrl activity may also prevent *M. genitalium* growth *in vivo*. Of note, it has been shown that Hrl from *M. genitalium* [32] and *M. pneumoniae* [64] elicit a prominent pro-inflammatory response and stimulate cytokine production through activation of NF-KB. Therefore, Hrl represents important virulence protein and a key therapeutic drug target of *M. genitalium*. In addition, Fur regulators are absent in eukaryotes and they also constitute attractive antibacterial targets. Several attempts have been made to develop therapies based on inhibition of Fur activity in other pathogens. Remarkably, several small peptides that inhibit Fur function have been shown to decrease pathogenic *E. coli* strain virulence in a fly infection model [65].

In summary, in this study, we describe the Fur regulon of *M. genitalium* and identify the proteins that are likely mediating nickel acquisition in this emerging human pathogen. Our results also suggest that zinc ions might regulate Fur activity in *M. genitalium*, although further work is necessary to determine the biochemical nature of this regulator. Furthermore, our results show a complex, multilayered transcriptional response to metal deprivation in this bacterium, suggesting a central role for metal regulatory systems in the survival of this urogenital pathogen within the host.

## Supplementary Material

Supplemental Material
