# Supplementary material for: Transcriptional response to metal starvation in the emerging pathogen Mycoplasma genitalium is mediated by Fur-dependent and –independent regulatory pathways
Source: Emerg Microbes Infect. 2019 Dec 20;9(1):5–19. doi: 10.1080/22221751.2019.1700762 (PMC6968530; doi:10.1080/22221751.2019.1700762)
Supplement: Supplemental Material [file TEMI_A_1700762_SM9981.zip › Supplemental_Information_final.docx]

**Construction of *M. genitalium* mutants**

***pΔMG_236***. This suicide plasmid was used to generate a *M. genitalium* *fur* null mutant by homologous recombination (HR). The MG_236 upstream region (UR) was amplified with the primers mg236 Up-F and mg236 Up-R and the downstream region (DR) was amplified with the mg236 Down-F and mg236 Down-R primers. The tetracycline resistance marker under the control of a constitutive promoter of *M. genitalium* (tetM438) was amplified with the primers Tc-F and Tc-R. Then, the UR and the tetM438 PCR products were joined by Splicing by Overlap Extension (SOE) PCR with the mg236 Up-F and Tc-R primers. Next, the product of the SOE-PCR and the DR were also joined using SOE-PCR with the mg236 Up-F and mg236 Down-R primers. The resulting PCR product was cloned into an *Eco*RV-digested pBE plasmid^1^. In order to reduce the polar effects derived from the insertion of the resistance marker, we cloned a transcription terminator after tetM438. This terminator sequence is present between the metal acquisition operon (MG_304-MG_302) and the *dnaK* gene (MG_305) and was identified using the TransTermHP software^2^. This transcriptional terminator was amplified using the TER305-F and TER305-R primers and digested with *Bam*HI. The plasmid was also digested with *Bam*HI and dephosphorylated to prevent self-ligation. Then, the terminator was cloned into the plasmid.

***pMTnCatMG_236***. This plasmid contains a minitransposon carrying a wild-type copy of the *fur* allele under its own promoter and was used to restore the wild-type phenotype of the *fur* mutant. The plasmid contains a chloramphenicol resistance marker. The MG_236 allele was amplified by PCR with the COMmg236-F (*Xba*I) and COMmg236-R (*Not*I), digested with *Xba*I and *Not*I and ligated into a digested pMTnCat plasmid^3^.

***pMTnWT149CatCh***. This plasmid carries a chloramphenicol acetyl transferase resistance marker fused to an mCherry tag (Cat:Ch) under the control of the promoter region of *hrl* (gcttatttagaaaaattcaaaataagcaaatTATAAT), which contains a putative *fur* box. The selectable marker fused to the fluorescent tag was amplified from a pCat:Ch plasmid^4^ with the wtMG149furbox-F and Ch-R primers. This PCR product was later digested with *Apa*I and *Xho*I and inserted in a similarly digested pMTnPac plasmid^5^, which carries a minitransposon with a puromycin resistance cassette.

***pMTnMUT149CatCh***. This plasmid carries a chloramphenicol acetyl transferase resistance marker fused to a mCherry tag under the control of a the *hrl* promoter with a scrambled sequence (gct**ac**t**a**tag**t**aaaat**a**caaa**tct**agcaaatTATAAT) at the putative *fur* box. This plasmid was constructed following the same steps as for the construction of the pMTnWT149CatCh, except that we used a different forward primer (mutMG149furbox-F) in order to alter the sequence of the putative *fur* box.

***pC1wtCatCh***. This plasmid was created to introduce a Cat:Ch fusion under the control of the *hrl* promoter region (gcttatttagaaaaattcaaaataagcaaatTATAAT) in the same exact chromosomic location as in the G37-Hrl_WT_:CatCh C1 strain, in order to compare the reporter fluorescence in a wild-type (G37 strain) and *fur* mutant background. We obtained the genomic DNA of the G37-Hrl_WT_:CatCh C1 strain and we determined the insertion point of the transposon by Sanger sequencing. Next, we amplified 1 kb upstream and downstream of the insertion point with the C1wt149CatCh-F and C1wt149CatCh-R primers. The PCR product including the upstream region, the Cat:Ch fusion under the control of the *hrl*  promoter region, and the downstream region was cloned into a *Eco*RV-digested pBE to create pC1wtCatCh.

***pC1mutCatCh***. This plasmid bears a copy of the Cat:Ch fusion under the control of the *hrl* promoter bearing the scrambled *fur* box sequence (gct**ac**t**a**tag**t**aaaat**a**caaa**tct**agcaaatTATAAT) in order to test the reporter fluorescence with an altered operator. The pC1wtCatCh plasmid was digested with with *Apa*I and *Xho*I to excise the cassette containing the Cat:Ch fusion under the control of the *hrl* promoter. The pMTnMUT149CatCh was digested similarly with *Apa*I and *Xho*I. Then, the construction bearing the Cat:Ch fusion regulated by the *hrl* promoter with the scrambled putative *fur* box was ligated into the backbone of the pC1wtCatCh plasmid.

**References**

1. Pich, O. Q., Burgos, R., Planell, R., Querol, E. & Piñol, J. Comparative analysis of antibiotic resistance gene markers in *Mycoplasma genitalium*: application to studies of the minimal gene complement. *Microbiology* **152**, 519–527 (2006).

2. Kingsford, C. L., Ayanbule, K. & Salzberg, S. L. Rapid, accurate, computational discovery of Rho-independent transcription terminators illuminates their relationship to DNA uptake. *Genome Biol.* **8**, R22 (2007).

3. Calisto, B. M. *et al.* The EAGR box structure: a motif involved in Mycoplasma motility. *Mol. Microbiol.* **86**, 382–393 (2012).

4. Torres-Puig, S., Broto, A., Querol, E., Piñol, J. & Pich, O. Q. A novel sigma factor reveals a unique regulon controlling cell-specific recombination in *Mycoplasma genitalium*. *Nucleic Acids Res.* **43**, 4923–4936 (2015).

5. Torres-Puig, S. *et al.* Activation of sigma20-dependent recombination and horizontal gene transfer in *Mycoplasma genitalium*. *DNA Res.* (2018). doi:10.1093/dnares/dsy011
