# Supplementary material for: Transcriptional response to metal starvation in the emerging pathogen Mycoplasma genitalium is mediated by Fur-dependent and –independent regulatory pathways
Source: Emerg Microbes Infect. 2019 Dec 20;9(1):5–19. doi: 10.1080/22221751.2019.1700762 (PMC6968530; doi:10.1080/22221751.2019.1700762)
Supplement: Supplemental Material [file TEMI_A_1700762_SM9981.zip › Supplemental_Tables_final.docx]

Table S1. Strains used in this study.

| **Strain name** | **Genotype** | **Reference** |
| --- | --- | --- |
| G37 | Wild-type | ATCC 33530 |
| *fur* | ∆MG_236::*tetM* | This work |
| *fur* COM | ∆MG_236::*tetM*, MG_438::Tn*Cm*MG_236 | This work |
| G37-Hrl_wt_:CatCh | Tn*Pac*Hrl_wt_:*cat*:*mcherry* | This work |
| G37-Hrl_mut_:CatCh | Tn*Pac*Hrl_mut_:*cat*:*mcherry* | This work |
| *fur*-Hrl_wt_:CatCh | Tn*Pac*Hrl_wt_:*cat*:*mcherry,* ∆MG_236::*tetM* | This work |
| *fur*-Hrl_mut_:CatCh | Tn*Pac*Hrl_mut_:*cat*:*mcherry,* ∆MG_236::*tetM* | This work |

Table S2. Plasmids used in this manuscript.

| **Plasmid name** | **Description** | **Source** |
| --- | --- | --- |
| pΔMG_236 | Suicide vector pBE (Pich *et al.*, 2006b) derivative used to create an MG_236 defective mutant. Selectable marker: tetracycline. | This work |
| pMTnCatMG_236 | Minitransposon miniTn4001 (Calisto *et al.*, 2012) derivative bearing an ectopic copy of MG_236 to complement the defective mutant. Selectable marker: chloramphenicol. | This work |
| pMTnWT149CatCh | Minitransposon miniTn4001 derivative carrying a chloramphenicol acetyl transferase resistance marker fused to an mCherry tag. Used to create the G37-Hrl_wt_:CatCh strain. Selectable marker: puromycin. | This work |
| pMTnMUT149CatCh | Minitransposon miniTn4001 derivative carrying a chloramphenicol acetyl transferase resistance marker fused to an mCherry tag. Selectable marker: puromycin. | This work |
| pC1wtCatCh | Suicide vector pBE derivative used to create the *fur*-Hrl_wt_:CatCh strain. Selectable marker: puromycin. | This work |
| pC1mutCatCh | Suicide vector pBE derivative used to create the G37-Hrl_mut_:CatCh and *fur*-Hrl_mut_:CatCh strains. Selectable marker: puromycin. | This work |

Table S3. Primers used in this study.

|  | **Primer Name** | **Sequence (5'-3')** |
| --- | --- | --- |
| **Mutants** | mg236 Up-F | CTTTGCAGGACAAAATGTTGC |
|  | mg236 Up-R | TAATTCTAAATACTAGAATTCACATAACTAGTTAGCATCTGG |
|  | mg236 Down-F | CAATAAAATAACTTAGGGATCCCCTTACCTTTAACAGATGGC |
|  | mg236 Down-R | CATCTTTTGTATCTACTAGTGC |
|  | TER305-F | GCGGGATCCCACAAGCAAAATAACCTGTTC |
|  | TER305-R | GCGGGATCCCTCGAGCTAAAAATCTGTTTTTTGGT |
|  | COMmg236-F(XbaI) | AGTTCTAGAGTTTGTTCTATCTAACTATTAAAGCAGTTAGAATTTGTTAGAATTACTTGTTTTAAAACTATGCTAACTAGTTATGTGAAGG |
|  | COMmg236-R(NotI) | AAAGCGGCCGCTTAATCATTGTTTATCTCACCCC |
|  | wtMG149furbox-F | ATTGGGCCCGCTTATTTAGAAAAATTCAAAATAAGCAAATTATAATTAGGTGTCTTTCTTTACTAAAAATATGGAGAAAAAAATCACTGG |
|  | mutMG149furbox-F | ATTGGGCCCGCTACTATAGTAAAATACAAATCTAGCAAATTATAATTAGGTGTCTTTCTTTACTAAAAATATGGAGAAAAAAATCACTGG |
|  | C1wt149CatCh-F | GATTGCTGCTCAATCAATTG |
|  | C1wt149CatCh-R | GACAACGCTTCAAAATTCACC |
|  | Cherry-R | AGTCTCGAGTTACTTGTACAGCTCGTCC |
|  | Tc-F | GAATTCTAGTATTTAGAATTAATAAAG |
|  | Tc-R | GGATCCCTAAGTTATTTTATTGAAC |
| **Screening** | mg236SCR-F | GTTGGTCAGATTATCTATAG |
|  | mg236SCR-R | CTCAACTTCCAAACAAAGAC |
|  | mg293SCR-F | GAAAACTAGCTAGTCAACAAG |
|  | mg293SCR-R | CGTCTTAAAAGTCTTCTTAC |
| **qRT-PCR** | RTPCRmg177-F | TGAGTGTCCAGCTGGTTTTG |
|  | RTPCRmg177-R | AACCGGGGAAAAGTTAGCAT |
|  | RTPCRmg418-F | TGTTGACGCTAGTGGTTTGG |
|  | RTPCRmg418-R | TTCCACCCATGTATTGAGAGTG |
|  | RTPCRmg430-F | GGAAGCAGTTGGATTGCCTA |
|  | RTPCRmg430-R | ATGCACTCCTCCATTGGAAA |
|  | RTPCRmg236-F | AATTGAACACCAAGATTGGC |
|  | RTPCRmg236-R | AGATAGATATGGTTATGCTC |
|  | RTPCRmg149-F | ACCAGGGATATGCACTAGCA |
|  | RTPCRmg149-R | TGCAACACTTTGGGTAGCTG |
|  | RTPCRmg304-F | GCTGATACACTCCACCAGGAA |
|  | RTPCRmg304-R | CAAGCAAAAACAGCACGTTG |
|  | RTPCRmCherry-F | GCCCCTAATGCAGAAGAAG |
|  | RTPCRmCherry-R | GTGTAGTCCTCGTTGTGGGA |
|  | RTPCRmg239-F | ATGCGAGAAAGCGCTAATGT |
|  | RTPCRmg239-R | TGCAGTTACCAAAGCAGCAC |
|  | RTPCRmg305-F | ATTTTGCTTGTGGACCTTGT |
|  | RTPCRmg305-R | GATATTAGCAAGTCCTGATG |
|  | RTPCRmg355-F | CACACCTGCTGGTGAAAATC |
|  | RTPCRmg355-R | CCAACTCCAGGTTCACCAAT |

|  | **Primer Name** | **Sequence (5'-3')** |
| --- | --- | --- |
| **Sequencing** | Fup-24 | CGCCAGGGTTTTCCCAGTCACGAC |
|  | Rup-24 | TCACACAGGAAACAGCTATGACCA |
|  | TetUp | TTCCTGCATCAACATGAG |
|  | TetDown | GTCGTCCAAATAGTCGGA |
|  | CmUp | CAACGGTGGTATATCCAG |
|  | CmDown | CAGTACTGCGATGAGTGGCA |
|  | PacUp | GTAGCTAATCTAACAGTAGG |
|  | PacDown | GTCCTAGAACTTGGTGTATG |
| **Primer extension reactions** | PEmg236(I) | [6-FAM]CACTTTAATAAAGCAATCCG |
|  | PEmg236(II) | [6-FAM]GGATCTACAAAGATGTTAATC |
|  | PEmg235(int) | [6-FAM]GTTAAGTCTCTGCCTATCTC |
|  | PEmg233 | [6-FAM]CAGCAAACAAAGCATGACCT |
|  | PEmg232 | [6-FAM]ATCACACAAACAACCTTAGC |

Table S4. Differentially expressed genes in *M. genitalium* upon metal starvation with 2,2’-bipyridyl identified by RNA-Seq analysis. Cut off log2±1.

| **Up-regulated** | | | | |
| --- | --- | --- | --- | --- |
| **Locus tag** | **Gene** | **Gene product** | **log2 Fold Change** | **p-value** |
| MG_355 | *clpB* | Chaperone protein ClpB | 3.84 | 2.09E-144 |
| MG_241 |  | Uncharacterized protein MG241 | 3.19 | 2.39E-98 |
| MG_239 | *lon* | Lon protease | 2.84 | 9.87E-211 |
| MG_242 |  | Uncharacterized protein MG242 | 2.66 | 8.28E-60 |
| MG_304 |  | ABC transporter ATP-binding protein MG304 | 2.59 | 6.49E-94 |
| MG_080 | *oppF* | Oligopeptide transport ATP-binding protein OppF | 2.47 | 2.23E-147 |
| MG_303 |  | ABC transporter ATP-binding protein MG303 | 2.42 | 7.56E-56 |
| MG_244 | *uvrD* | DNA helicase II homolog | 2.33 | 5.32E-38 |
| MG_302 |  | Uncharacterized protein | 2.22 | 5.71E-21 |
| MG_079 | *oppD* | Oligopeptide transport ATP-binding protein OppD | 2.16 | 1.12E-79 |
| MG_524 |  | Uncharacterized protein MG384.1 | 2.14 | 1.71E-13 |
| MG_078 | *oppC* | Oligopeptide transport system permease protein OppC | 2.12 | 2.60E-50 |
| MG_149 | *hrl* | Uncharacterized lipoprotein MG149 | 2.12 | 3.73E-113 |
| MG_305 | *dnaK* | Chaperone protein DnaK | 2.01 | 8.67E-57 |
| MG_439 |  | Uncharacterized lipoprotein MG439 | 1.94 | 5.13E-31 |
| MG_077 | *oppB* | Oligopeptide transport system permease protein OppB | 1.93 | 8.60E-51 |
| MG_316 |  | Uncharacterized protein MG316 | 1.85 | 7.12E-08 |
| MG_192.1 |  | Uncharacterized small protein MG192.1 | 1.74 | 6.27E-20 |
| MG_321 |  | Uncharacterized lipoprotein MG321 | 1.65 | 2.68E-55 |
| MG_339 | *recA* | Protein RecA | 1.64 | 2.94E-43 |
| MG_033 | *glpF* | Glycerol uptake facilitator protein | 1.60 | 1.21E-47 |
| MG_083 | *pth* | Peptidyl-tRNA hydrolase | 1.53 | 2.49E-51 |
| MG_084 | *tilS* | tRNA(Ile)-lysidine synthase | 1.50 | 9.12E-44 |
| MG_085 | *hprK* | HPr kinase/phosphorylase | 1.49 | 5.32E-44 |
| MG_384 | *obg* | GTPase Obg | 1.49 | 2.56E-37 |
| MG_389 |  | Uncharacterized protein MG389 | 1.47 | 5.86E-05 |
| MG_390 |  | ABC transporter ATP-binding protein MG390 | 1.37 | 5.37E-34 |
| MG_282 | *greA* | Transcription elongation factor GreA | 1.36 | 4.84E-63 |
| MG_478 |  | Uncharacterized protein MG149.1 | 1.32 | 5.04E-17 |
| MG_521 |  | Uncharacterized protein MG350.1 | 1.31 | 0.0178199 |
| MG_081 | *rplK* | 50S ribosomal protein L11 | 1.31 | 9.44E-35 |
| MG_098 |  | Uncharacterized protein MG098 | 1.30 | 1.23E-22 |
| MG_441 |  | Uncharacterized protein MG441 | 1.29 | 0.00079279 |
| MG_245 |  | 5-formyltetrahydrofolate cyclo-ligase | 1.28 | 9.94E-06 |
| MG_012 |  | Uncharacterized protein MG012 | 1.25 | 6.17E-10 |
| MG_206 | *uvrC* | UvrABC system protein C | 1.21 | 6.69E-24 |
| MG_074 |  | Uncharacterized protein MG074 | 1.19 | 0.00038207 |
| MG_082 | *rplA* | 50S ribosomal protein L1 | 1.16 | 4.78E-26 |
| MG_385 |  | Uncharacterized protein MG385 | 1.14 | 4.81E-18 |
| MG_237 |  | Uncharacterized protein MG237 | 1.13 | 7.28E-23 |
| MG_064 |  | Uncharacterized ABC transporter permease MG064 | 1.10 | 4.54E-24 |
| MG_075 |  | Uncharacterized protein MG075 | 1.08 | 5.38E-35 |
| MG_365 | *fmt* | Methionyl-tRNA formyltransferase | 1.07 | 5.47E-08 |
| MG_246 |  | Putative phosphatase/phosphodiesterase MG246 | 1.06 | 1.08E-13 |
| MG_236 | *fur* | Ferric uptake regulation protein | 1.03 | 5.40E-15 |
| MG_358 | *ruvA* | Holliday junction ATP-dependent DNA helicase RuvA | 1.03 | 3.31E-13 |

| **Down-regulated** | | | | |
| --- | --- | --- | --- | --- |
| **Locus tag** | **Gene** | **Gene product** | **log2 Fold Change** | **p-value** |
| MG_189 |  | ABC transporter permease protein MG189 | -1.98 | 6.16E-46 |
| MG_188 |  | ABC transporter permease protein MG188 | -1.73 | 1.85E-33 |
| MG_511 |  | tRNA-Val | -1.42 | 2.68E-04 |
| MG_489 |  | tRNA-Asp | -1.39 | 9.82E-05 |
| MG_512 |  | tRNA-Thr | -1.35 | 1.59E-05 |
| MG_485 |  | tRNA-Met | -1.34 | 3.23E-06 |
| MG_112 | *rpe* | Ribulose-phosphate 3-epimerase | -1.33 | 8.21E-15 |
| MG_501 |  | tRNA-Lys | -1.31 | 3.06E-04 |
| MG_434 | *pyrH* | Uridylate kinase | -1.31 | 1.68E-30 |
| MG_350 |  | Uncharacterized protein MG350 | -1.29 | 1.77E-14 |
| MG_187 |  | ABC transporter ATP-binding protein MG187 | -1.28 | 1.20E-29 |
| MG_483 |  | tRNA-Cys | -1.28 | 1.33E-04 |
| MG_111 | *pgi* | Glucose-6-phosphate isomerase | -1.27 | 8.22E-36 |
| MG_453 | *galU* | Glucose-1-phosphate uridylyltransferase | -1.23 | 5.48E-20 |
| MG_500 |  | tRNA-Leu | -1.22 | 1.50E-04 |
| MG_132 |  | Uncharacterized HIT-like protein MG132 | -1.2 | 7.65E-14 |
| MG_484 |  | tRNA-Pro | -1.19 | 3.72E-03 |
| MG_324 | *pepP* | Putative Xaa-Pro aminopeptidase | -1.17 | 4.16E-21 |
| MG_526 |  | ABC transporter ATP-binding protein MG468.1 | -1.13 | 6.15E-18 |
| MG_510 |  | tRNA-Thr | -1.13 | 5.44E-04 |
| MG_513 |  | tRNA-Glu | -1.11 | 1.26E-03 |
| MG_186 |  | Uncharacterized lipoprotein MG186 | -1.11 | 4.12E-11 |
| MG_507 |  | tRNA-Ser | -1.09 | 1.21E-03 |
| MG_515 |  | Uncharacterized protein MG323.1 | -1.08 | 4.21E-17 |
| MG_024 | *ychF* | Ribosome-binding ATPase YchF | -1.08 | 1.34E-19 |
| MG_072 | *secA* | Protein translocase subunit SecA | -1.08 | 5.23E-22 |
| MG_514 |  | tRNA-Asn | -1.07 | 1.32E-04 |
| MG_488 |  | tRNA-Met | -1.07 | 1.66E-03 |
| MG_023 | *fba* | Fructose-bisphosphate aldolase | -1.07 | 3.76E-20 |
| MG_506 |  | tRNA-Ser | -1.02 | 2.83E-02 |
| MG_494 |  | Uncharacterized protein MG255.1 | -1.02 | 3.37E-03 |
| MG_278 | *relA* | Guanosine-3',5'-bis(diphosphate) 3'-pyrophosphohydrolase | -1.02 | 3.18E-15 |
| MG_190 | *nrnA* | Bifunctional oligoribonuclease and PAP phosphatase NrnA | -1.02 | 9.17E-23 |
| MG_435 | *frr* | Ribosome-recycling factor | -1.01 | 1.05E-11 |
| MG_262 | *polA* | 5'-3' exonuclease | -1 | 2.08E-07 |

Table S5. Differentially expressed genes in a *M. genitalium* *fur* mutant and the corresponding complemented strain derivative compared to the wild-type strain identified by RNA-Seq analysis. Cut off log2±1.

|  |  |  | *fur* | | *fur* COM | |
| --- | --- | --- | --- | --- | --- | --- |
| **Locus tag** | **Gene** | **Gene product** | **log2 fold change** | **p-value** | **log2 fold change** | **p-value** |
| MG_149 | *hrl* | Uncharacterized lipoprotein MG149 | 2.45 | 2.09E-144 | -0.95 | 1.87E-11 |
| MG_304 |  | ABC transporter ATP-binding protein MG304 | 2.2 | 9.87E-211 | -0.35 | 0.10 |
| MG_303 |  | ABC transporter ATP-binding protein MG303 | 2.06 | 6.49E-94 | -0.97 | 6.86E-05 |
| MG_302 |  | Uncharacterized protein MG302 | 1.45 | 7.56E-56 | -0.37 | 0.47 |
| MG_236 | *fur* | Ferric uptake regulation protein | -5.77 | 3.13E-41 | 3.53 | 3.02E-293 |
| MG_237 |  | Uncharacterized protein MG237 | -1.25 | 1.50E-05 | -1.22 | 4.28E-14 |

Table S6. Differentially expressed genes in a *M. genitalium* *fur* mutant upon metal deprivation with 2,2’-bipyridyl identified by RNA-Seq analysis. Cut off log2±1.

| **Up-regulated** | | | | |
| --- | --- | --- | --- | --- |
| **Locus tag** | **Gene** | **Gene product** | **log2 Fold Change** | **p-value** |
| MG_355 | *clpB* | Chaperone protein ClpB | 4.33 | 2.35E-202 |
| MG_241 |  | Uncharacterized protein MG241 | 3.69 | 3.97E-99 |
| MG_239 | *lon* | Lon protease | 3.02 | 3.42E-66 |
| MG_079 | *oppD* | Oligopeptide transport ATP-binding protein OppD | 2.77 | 2.38E-90 |
| MG_078 | *oppC* | Oligopeptide transport system permease protein OppC | 2.66 | 6.15E-63 |
| MG_080 | *oppF* | Oligopeptide transport ATP-binding protein OppF | 2.58 | 7.58E-57 |
| MG_083 | *pth* | Peptidyl-tRNA hydrolase | 2.57 | 1.53E-29 |
| MG_242 |  | Uncharacterized protein MG242 | 2.49 | 3.24E-33 |
| MG_081 | *rplK* | 50S ribosomal protein L11 | 2.40 | 8.93E-31 |
| MG_082 | *rplA* | 50S ribosomal protein L1 | 2.37 | 1.87E-36 |
| MG_244 | *uvrD* | DNA helicase II homolog | 2.36 | 8.01E-51 |
| MG_077 | *oppB* | Oligopeptide transport system permease protein OppB | 2.17 | 8.69E-64 |
| MG_305 | *dnaK* | Chaperone protein DnaK | 2.09 | 6.78E-35 |
| MG_321 |  | Uncharacterized lipoprotein MG321 | 2.07 | 4.19E-48 |
| MG_339 | *recA* | Protein RecA | 2.05 | 1.79E-22 |
| MG_478 |  | Uncharacterized protein MG149.1 | 1.91 | 1.78E-17 |
| MG_084 | *tilS* | tRNA(Ile)-lysidine synthase | 1.88 | 1.64E-38 |
| MG_012 |  | Uncharacterized protein MG012 | 1.78 | 3.20E-15 |
| MG_282 | *greA* | Transcription elongation factor GreA | 1.77 | 8.59E-20 |
| MG_174 | *rpmJ* | 50S ribosomal protein L36 | 1.73 | 2.11E-09 |
| MG_246 |  | Putative phosphatase/phosphodiesterase MG246 | 1.72 | 4.62E-16 |
| MG_384 | *obg* | GTPase Obg | 1.70 | 1.35E-17 |
| MG_439 |  | Uncharacterized lipoprotein MG439 | 1.70 | 3.08E-12 |
| MG_085 | *hprK* | HPr kinase/phosphorylase | 1.61 | 3.06E-25 |
| MG_098 |  | Uncharacterized protein MG098 | 1.58 | 1.45E-29 |
| MG_358 | *ruvA* | Holliday junction ATP-dependent DNA helicase RuvA | 1.57 | 3.59E-10 |
| MG_393 | *groES* | 10 kDa chaperonin | 1.56 | 1.54E-12 |
| MG_206 | *uvrC* | UvrABC system protein C | 1.54 | 6.62E-14 |
| MG_353 |  | Uncharacterized protein MG353 | 1.52 | 1.18E-19 |
| MG_142 | *infB* | Translation initiation factor IF-2 | 1.46 | 4,36E-09 |
| MG_521 |  | Uncharacterized protein MG350.1 | 1.44 | 1.90E-02 |
| MG_392 | *groL* | 60 kDa chaperonin | 1.31 | 3.49E-19 |
| MG_245 |  | 5-formyltetrahydrofolate cyclo-ligase | 1.31 | 0.00039983 |
| MG_149 | *hrl* | Uncharacterized lipoprotein MG149 | 1.30 | 4.13E-14 |
| MG_340 | *rpoC* | DNA-directed RNA polymerase subunit beta' | 1.28 | 6.92E-14 |
| MG_440 |  | Uncharacterized protein MG440 | 1.28 | 4,49E-12 |
| MG_359 | *ruvB* | Holliday junction ATP-dependent DNA helicase RuvB | 1.24 | 2.22E-09 |
| MG_192.1 |  | Uncharacterized small protein MG192.1 | 1.21 | 6.35E-07 |
| MG_179 | *ecfA1* | Energy-coupling factor transporter ATP-binding protein EcfA1 | 1.19 | 2.03E-09 |
| MG_173 | *infA* | Translation initiation factor IF-1 | 1.17 | 3.20E-06 |
| MG_238 | *tig* | Trigger factor | 1.15 | 5.74E-08 |
| MG_058 | *prs* | Ribose-phosphate pyrophosphokinase | 1.13 | 2.72E-09 |
| MG_473 | *rpmG2* | 50S ribosomal protein L33 2 | 1.12 | 7.99E-07 |
| MG_161 | *rplN* | 50S ribosomal protein L14 | 1.12 | 3.06E-05 |
| MG_011 |  | Uncharacterized protein MG011 | 1.06 | 1.93E-04 |
| MG_164 | *rpsN* | 30S ribosomal protein S14 type Z | 1.06 | 1.16E-04 |
| MG_100 | *gatB* | Aspartyl/glutamyl-tRNA(Asn/Gln) amidotransferase subunit B | 1.05 | 2.17E-12 |
| MG_237 |  | Uncharacterized protein MG237 | 1.05 | 4.53E-06 |
| MG_158 | *rplP* | 50S ribosomal protein L16 | 1.05 | 1.53E-05 |
| MG_180 | *ecfA2* | Energy-coupling factor transporter ATP-binding protein EcfA2 | 1.03 | 1.41E-10 |
| MG_316 |  | Uncharacterized protein MG316 | 1.02 | 9.22E-02 |
| MG_160 | *rpsQ* | 30S ribosomal protein S17 | 1.00 | 3.38E-04 |

| **Down-regulated** | | | | |
| --- | --- | --- | --- | --- |
| **Locus tag** | **Gene** | **Gene product** | **log2 Fold Change** | **p-value** |
| MG_452 |  | Uncharacterized protein MG452 | -2.31 | 3.56E-09 |
| MG_505 |  | Putative pre-16S rRNA nuclease | -1.93 | 3.97E-03 |
| MG_007 |  | Uncharacterized protein MG007 | -1.91 | 1.05E-03 |
| MG_494 |  | Uncharacterized protein MG255.1 | -1.87 | 4.82E-04 |
| MG_522 | *rpsT* | 30S ribosomal protein S20 | -1.86 | 6.78E-06 |
| MG_039 |  | Uncharacterized protein MG039 | -1.73 | 9.02E-18 |
| MG_189 |  | ABC transporter permease protein MG189 | -1.71 | 4.90E-21 |
| MG_453 | *galU* | Glucose-1-phosphate uridylyltransferase | -1.7 | 4.92E-16 |
| MG_350 |  | Uncharacterized protein MG350 | -1.68 | 9.45E-10 |
| MG_409 |  | Uncharacterized protein MG409 | -1.53 | 1.42E-04 |
| MG_435 | *frr* | Ribosome-recycling factor | -1.51 | 1.39E-07 |
| MG_188 |  | ABC transporter permease protein MG188 | -1.5 | 5.10E-16 |
| MG_437 | *cdsA* | Phosphatidate cytidylyltransferase | -1.48 | 4.61E-11 |
| MG_010 |  | Uncharacterized protein MG010 | -1.48 | 1.24E-02 |
| MG_001 | *dnaN* | Beta sliding clamp | -1.44 | 9.69E-03 |
| MG_380 | *rsmG* | Ribosomal RNA small subunit methyltransferase G | -1.42 | 9.40E-03 |
| MG_515 |  | Uncharacterized protein MG323.1 | -1.41 | 3.17E-07 |
| MG_526 |  | ABC transporter ATP-binding protein MG468.1 | -1.41 | 3.02E-12 |
| MG_071 | *pacL* | Cation-transporting P-type ATPase | -1.38 | 1.23E-14 |
| MG_429 | *ptsI* | Phosphoenolpyruvate-protein phosphotransferase | -1.34 | 1.89E-19 |
| MG_255 |  | Uncharacterized protein MG255 | -1.31 | 5.66E-14 |
| MG_495 |  | tRNA-Arg | -1.3 | 1.74E-04 |
| MG_431 | *tpiA* | Triosephosphate isomerase | -1.26 | 1.96E-09 |
| MG_428 |  | Alternative sigma factor σ20 | -1.26 | 4.96E-05 |
| MG_248 |  | tRNA methyltransferase MG248 | -1.25 | 3.30E-06 |
| MG_262 | *polA* | 5'-3' exonuclease | -1.25 | 8.83E-08 |
| MG_325 | *rpmG1* | 50S ribosomal protein L33 1 | -1.24 | 1.07E-07 |
| MG_112 | *rpe* | Ribulose-phosphate 3-epimerase | -1.24 | 1.25E-05 |
| MG_438 |  | Type-1 restriction enzyme specificity protein MG438 | -1.24 | 6.66E-14 |
| MG_110 | *rsgA* | Small ribosomal subunit biogenesis GTPase RsgA | -1.22 | 1.95E-04 |
| MG_111 | *pgi* | Glucose-6-phosphate isomerase | -1.2 | 1.11E-11 |
| MG_422 |  | Uncharacterized protein MG422 | -1.2 | 5.02E-03 |
| MG_447 |  | Uncharacterized protein MG447 | -1.19 | 1.45E-03 |
| MG_507 |  | tRNA-Ser | -1.19 | 2.37E-05 |
| MG_470 |  | ParA family protein MG470 | -1.18 | 9.71E-11 |
| MG_370 |  | Uncharacterized RNA pseudouridine synthase MG370 | -1.16 | 5.53E-03 |
| MG_296 |  | Uncharacterized protein MG296 | -1.14 | 2.84E-03 |
| MG_520 |  | tRNA-Leu | -1.12 | 1.12E-04 |
| MG_485 |  | tRNA-Met | -1.12 | 1.50E-04 |
| MG_463 | *rsmA* | Ribosomal RNA small subunit methyltransferase A | -1.12 | 2.09E-04 |
| MG_074 |  | Uncharacterized protein MG074 | -1.12 | 1.88E-03 |
| MG_123 |  | Uncharacterized protein MG123 | -1.11 | 1.79E-07 |
| MG_047 | *metK* | S-adenosylmethionine synthase | -1.09 | 2.61E-10 |
| MG_135 |  | Uncharacterized protein MG135 | -1.07 | 1.21E-05 |
| MG_240 |  | Uncharacterized protein MG240 | -1.04 | 7.16E-06 |
| MG_140 |  | Uncharacterized ATP-dependent helicase MG140 | -1.04 | 2.37E-15 |
| MG_132 |  | Uncharacterized HIT-like protein MG132 | -1.04 | 5.33E-07 |
| MG_372 | *thiI* | Probable tRNA sulfurtransferase | -1.04 | 7.28E-05 |
| MG_324 | *pepP* | Putative Xaa-Pro aminopeptidase | -1.02 | 2.60E-13 |
| MG_382 | *udk* | Uridine kinase | -1.01 | 2.31E-03 |
| MG_343 |  | Uncharacterized protein MG343 | -1.01 | 1.21E-02 |

Table S7. Differentially expressed proteins observed in the DIGE proteomic analysis of the fur mutant.

| Spot # | Id Score^1^ | #Peptides^1^ | % SC^1^ | Accession^2^ | Entry Name^2^ | Protein | Locus | Gene | log2 FC | | p-value |
| --- | --- | --- | --- | --- | --- | --- | --- | --- | --- | --- | --- |
| 1766 | 460 | 10 | 54.1 | P47648 | MSRA_MYCGE | Peptide methionine sulfoxide reductase MsrA | MG408 | *msrA* | | 2.49 | 1.60E-02 |
| 2076 | 314 | 6 | 28.0 | P47648 | MSRA_MYCGE | Peptide methionine sulfoxide reductase MsrA | MG408 | *msrA* | | 2.42 | 6.00E-03 |
| 2065 | 116 | 2 | 12.1 | P47648 | MSRA_MYCGE | Peptide methionine sulfoxide reductase MsrA | MG408 | *msrA* | | 2.23 | 3.00E-03 |
| 2118 | 167 | 5 | 14.4 | P47515 | ODPB_MYCGE | Pyruvate dehydrogenase E1 component E1, subunit beta | MG273 | *pdhB* | | 2.64 | 2.26E-04 |
| 1771 | 84 | 3 | 23.2 | P47334 | RS7_MYCGE | 30S ribosomal protein S7 | MG088 | *rpsG* | | 2.54 | 2.00E-03 |
| 2143 | 163 | 4 | 26.5 | P47656 | RS9_MYCGE | 30S ribosomal protein S9 | MG417 | *rpsI* | | 2.19 | 2.60E-02 |
| 1838 | 114 | 3 | 21.2 | P47656 | RS9_MYCGE | 30S ribosomal protein S9 | MG417 | *rpsI* | | 2.08 | 2.00E-03 |
| 2064 | 235 | 5 | 14.6 | P47395 | Y149_MYCGE | Uncharacterized lipoprotein MG149 - Histidine-rich lipoprotein | MG149 | *hrl* | | 2.60 | 3.18E-04 |
| 1808 | 112 | 2 | 6.4 | P47395 | Y149_MYCGE | Uncharacterized lipoprotein MG149 - Histidine-rich lipoprotein | MG149 | *hrl* | | 2.22 | 1.30E-02 |
| 1810 | 122 | 2 | 1.8 | P47580 | Y338_MYCGE | Uncharacterized lipoprotein MG338 | MG338 | MG_338 | | 2.60 | 3.00E-03 |
| 2075 | 192 | 4 | 3.1 | P47580 | Y338_MYCGE | Uncharacterized lipoprotein MG338 | MG338 | MG_338 | | 2.45 | 6.00E-03 |
| 1918 |  |  |  |  |  |  |  |  | | 3.02 | 8.45E-04 |
| 2145 |  |  |  |  |  |  |  |  | | 1.87 | 1.00E-02 |
| 2111 |  |  |  |  |  |  |  |  | | 1.65 | 5.00E-03 |
| 1881 |  |  |  |  |  |  |  |  | | 1.58 | 4.50E-02 |
| 2138 |  |  |  |  |  |  |  |  | | 1.47 | 1.20E-02 |

1 Protein Identification Score in Mascot MSMS search, number of unique peptides identified and % sequence coverage.

2 UniProt/SwissProt identifiers

Table S8. Insertion site of the TnPacHrl_wt_:*cat*:*mcherry* transposon in the genome of two different G37-Hrlwt:CatCh clones.

| **G37-Hrl_wt_CatCh clone** | **Fluorescence** | **Insertion site (bp)** | **Gene truncated** |
| --- | --- | --- | --- |
| C1 | Marginal | 430390 | None |
| C5 | Marginal | 72884 | *fruK* |

Table S9. Concentration of some transition metals in the SP-4 medium determined by ICP-MS analysis (n=10).

| **Element** | **Mean (µg/L)** | **SD** |
| --- | --- | --- |
| Mn | 27.1 | 2.47 |
| Fe | 691.3 | 33.55 |
| Co | 9.72 | 0.64 |
| Ni | 5.52 | 1.11 |
| Cu | 33.7 | 3.83 |
| Zn | 948.5 | 39.31 |

SD: Standard Deviation
