# Supplementary figures and images for: Transcriptional response to metal starvation in the emerging pathogen Mycoplasma genitalium is mediated by Fur-dependent and –independent regulatory pathways
Source: Emerg Microbes Infect. 2019 Dec 20;9(1):5–19. doi: 10.1080/22221751.2019.1700762 (PMC6968530; doi:10.1080/22221751.2019.1700762)

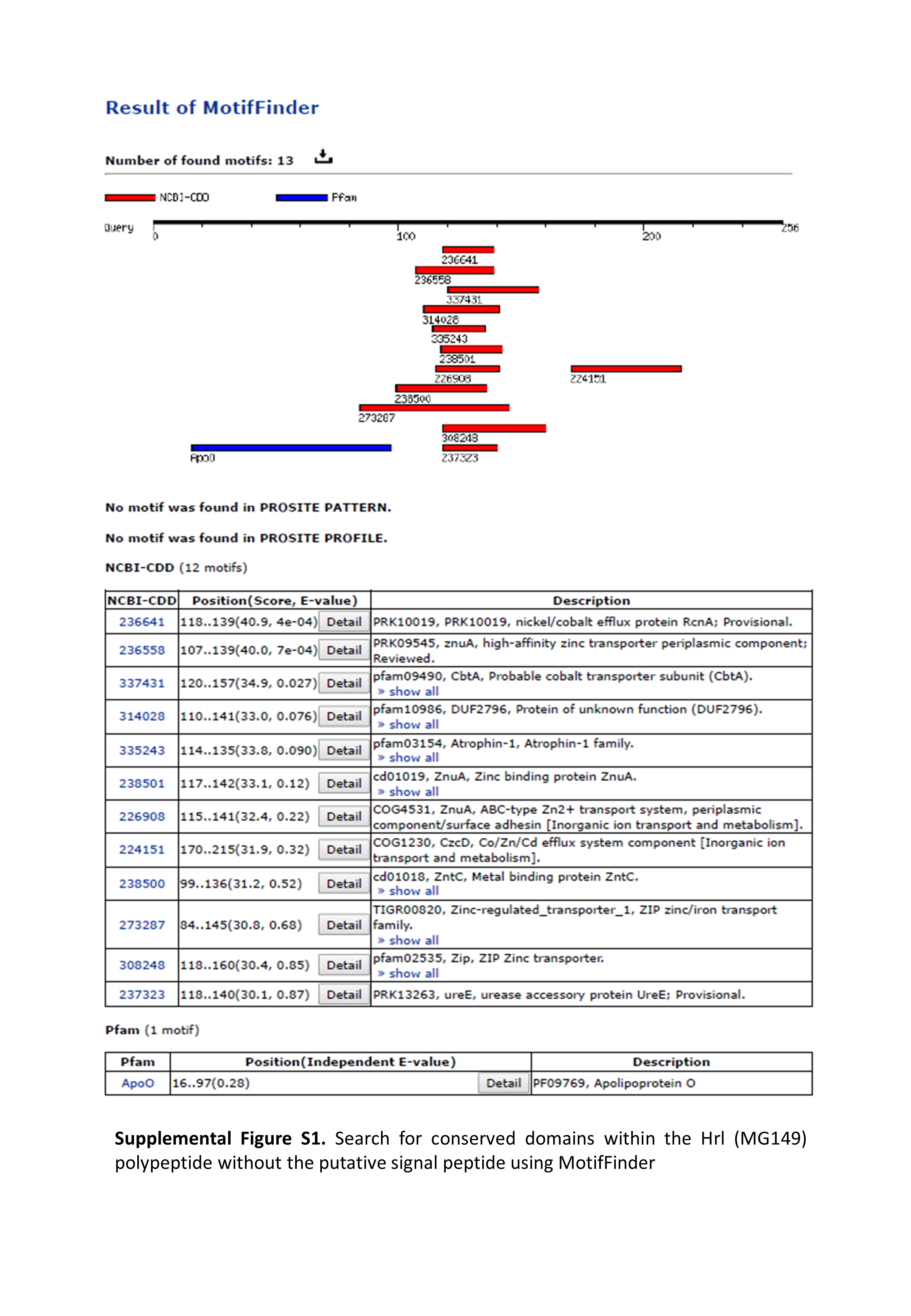

Supplement: Supplemental Material [file TEMI_A_1700762_SM9981.zip › Figure_S1_final.tif]
